# Machine-learning-based integrative –‘omics analyses reveal immunologic and metabolic dysregulation in environmental enteric dysfunction

**DOI:** 10.1016/j.isci.2024.110013

**Published:** 2024-05-17

**Authors:** Fatima Zulqarnain, Xueheng Zhao, Kenneth D.R. Setchell, Yash Sharma, Phillip Fernandes, Sanjana Srivastava, Aman Shrivastava, Lubaina Ehsan, Varun Jain, Shyam Raghavan, Christopher Moskaluk, Yael Haberman, Lee A. Denson, Khyati Mehta, Najeeha T. Iqbal, Najeeb Rahman, Kamran Sadiq, Zubair Ahmad, Romana Idress, Junaid Iqbal, Sheraz Ahmed, Aneeta Hotwani, Fayyaz Umrani, Beatrice Amadi, Paul Kelly, Donald E. Brown, Sean R. Moore, Syed Asad Ali, Sana Syed

**Affiliations:** 1 University of Virginia, Charlottesville, VA, USA; 2 Cincinnati Children’s Hospital Medical Center, University of Cincinnati School of Medicine, Cincinnati, OH, USA; 3 Aga Khan University, Karachi, Pakistan; 4 University Teaching Hospital, Lusaka, Zambia; 5 Queen Mary University of London, London, UK

**Keywords:** Gastroenterology, Medical imaging, Metabolic flux analysis, Lipidomics, Transcriptomics, Machine learning

## Abstract

Environmental enteric dysfunction (EED) is a subclinical enteropathy challenging to diagnose due to an overlap of tissue features with other inflammatory enteropathies. EED subjects (*n* = 52) from Pakistan, controls (*n* = 25), and a validation EED cohort (*n* = 30) from Zambia were used to develop a machine-learning-based image analysis classification model. We extracted histologic feature representations from the Pakistan EED model and correlated them to transcriptomics and clinical biomarkers. *In-silico* metabolic network modeling was used to characterize alterations in metabolic flux between EED and controls and validated using untargeted lipidomics. Genes encoding beta-ureidopropionase, CYP4F3, and epoxide hydrolase 1 correlated to numerous tissue feature representations. Fatty acid and glycerophospholipid metabolism-related reactions showed altered flux. Increased phosphatidylcholine, lysophosphatidylcholine (LPC), and ether-linked LPCs, and decreased ester-linked LPCs were observed in the duodenal lipidome of Pakistan EED subjects, while plasma levels of glycine-conjugated bile acids were significantly increased. Together, these findings elucidate a multi-omic signature of EED.

## Highlights

• Multiomic approach to study environmental enteric dysfunction
• Combines machine-learning-based image-analysis, metabolic modeling and lipidomics

## Graphical abstract

**Figure:**
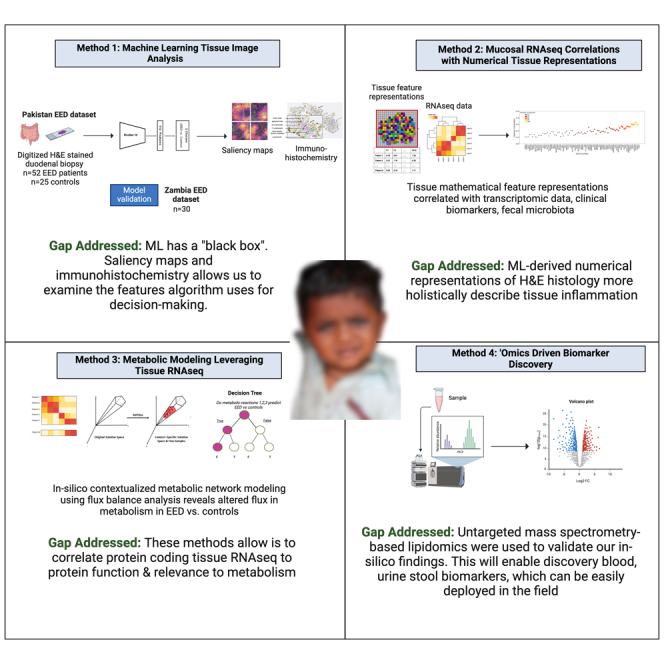

## Introduction

Environmental enteric dysfunction (EED) is a subclinical enteropathy highly prevalent in low-and-middle-income countries (LMICs) characterized by small intestinal structure and function alterations, leading to undernutrition, poor oral vaccine immunogenicity, and cognitive dysfunction.^1^^,^^2^^,^^3^ EED is believed to result from the cumulative effects of marginal diets, unsanitary environments, and microbial dysbiosis.^4^^,^^5^ The current mainstay of diagnosis remains endoscopy with biopsy and subsequent histologic evaluation,^6^ as there is no universally accepted case definition of EED nor any sensitive or specific biomarkers validated for its diagnosis. Compounding this diagnostic difficulty, the histopathologic examination of EED tissue reveals non-specific features of intestinal inflammation, including small intestinal villus atrophy, crypt hyperplasia, and inflammation.^1^^,^^6^^,^^7^^,^^8^^,^^9^ In recent years, individual and multicounty studies have aimed to understand better the pathophysiology, predictors, biomarkers, and potential management strategies of EED^4^^,^^10^^,^^11^^,^^12^ These studies have yielded substantial data, including histopathologic images, RNA sequence data, and lipidomic profiles, presenting a unique opportunity to integrate these multiple modalities of data to gain novel insights into EED pathogenesis.

Machine learning-based methods allow us to use algorithms and statistical models to analyze and draw inferences from patterns in complex multimodal data.^13^^,^^14^^,^^15^^,^^16^ Integrative analyses of high-throughput biologic data, including genomics, lipidomics, and proteomics, have previously been used with considerable success to analyze histopathologic images in cancer subtype classification and grading, quantifying tumor immune infiltration, and cell segmentation in eosinophilic esophagitis.^17^^,^^18^^,^^19^ Integrating multimodal data with cutting-edge machine learning approaches can bridge the gap between our understanding of the expression of certain genes, their functions, and specific phenotypes observable in histopathology to greatly improve EED evaluation by accurately characterizing distinct yet morphologically related diseases.

In this work, we developed a machine-learning-based image analysis platform to understand the microscopic structural differences between subjects with EED from Pakistan and controls from the United States of America (USA). Our findings were validated through the visualization of saliency maps, and targeted immunohistochemical quantification. We further validated our machine learning model in a geographically distinct dataset from Zambia. Next, we linked changes in molecular expression data to tissue-level changes to reveal novel tissue and genomic features, which may serve as specific biomarkers of EED. Next, we correlated hematoxylin and eosin (H&E)-stained histopathologic images and clinical biomarkers of patients with EED from Pakistan with gene expression data. Finally, we used contextualized metabolic network modeling to examine the functional differences in metabolism in subjects with or without EED from both Pakistan and Zambia and then validated these signatures using untargeted mass spectrometry-based lipidomic assays of duodenal aspirate and plasma samples from Pakistan. Combined, these findings may enable us to elucidate potential genomic and biochemical markers of EED.

## Results

### Detailed demographic descriptions of the study cohorts

A total of 416 children were enrolled in the Study of Environmental Enteropathy and Malnutrition (SEEM) at a median enrollment time of 5 days after birth, with *n* = 365 malnourished cases and *n* = 51 age-matched well-nourished controls.^10^^,^^20^ Participants underwent anthropometric measurement monthly, from birth to 24 months, and the participants also received education on optimizing nutrition. At 9 months, blood, urine, and fecal samples were collected from the participants. Participants with wasting (weight for height, or WHZ score < −2) at the 9-month visit were provided with additional nutritional intervention in the form of high-calorie AchaMum therapeutic food until the age of 12 months (*n* = 189). Of this sub-cohort, *n* = 63 patients with wasting refractory to nutritional intervention underwent esophagogastroduodenoscopy. Duodenal samples from this cohort were embedded in paraffin, stained with hematoxylin and eosin (H&E), and evaluated by two trained pathologists at the Aga Khan University in Karachi, Pakistan. An additional biopsy was obtained for RNA sequencing for *n* = 52 patients. In this article, our Pakistan cohort comprises *n* = 52 patients with both H&E-stained slides and RNA sequencing data from the Pakistan cohort.^20^

The Pakistan EED cohort had a median age of 1.7 years (IQR: 1.4, 1.9 years) and a median weight-for-height *Z* score of −2.2 (IQR: −2.6, −1.8). At Cincinnati Children’s Hospital, USA, *n* = 25 pediatric controls with no histopathologic abnormalities were enrolled as a comparison group. This latter cohort had a median age of 5.4 years (IQR: 3.8, 6.8 years). Detailed anthropometric data at the time of endoscopy is detailed for each cohort in Table 1. Data regarding detailed blood biomarkers, Environmental Enteric Dysfunction Biopsy Initiative (EEDBI) scores, and the measurement of enteropathogen burden in duodenal aspirate using quantitative polymerase chain reactions in the Pakistan cohort, collected as part of the SEEM study are summarized in Table 2.^20^

**Table 1:** Demographic description of clinical cohorts

Site	EED (Pakistan^a^) (*n* = 52)	EED (Zambia^b^) (*n* = 30)	Controls (USA^c^) (*n* = 25)
Sex (% Female)	31%	50%	44%
Median age at time of endoscopy (years, [IQR])	1.7 [1.4, 1.9]	1.1 [0.4, 1.3]	5.4 [3.8, 6.8]
Anthropometric measures at the time of endoscopy			
HAZ (median, [IQR])	−3.2 [-3.6, −2.3]	−3.3 [-3.9, −2.8]	0.1 [-0.5, 0.8]
WAZ (median, [IQR])	−2.9 [-3.5, −2.6]	−2.3 [-2.7, −1.8]	−0.1 [-1.1, 0.8]
WHZ (median, [IQR])	−2.2[(-2.6, −1.8]	−0.7 [-1.3, −0.2]	N/A

EED, environmental enteric dysfunction; HAZ, Height-for-age Z score; WAZ, Weight-for-age Z score; WHZ, Weight-for-height Z score; IQR, interquartile range; N/A, not available.

a Subjects in our Pakistan EED cohort were previously enrolled as part of the Study of Environmental Enteropathy and Malnutrition (SEEM) study in Matiari, Pakistan.

b Subjects in our Zambia EED cohort were previously enrolled as part of the Biomarkers of Environmental Enteropathy in Children (BEECH) study in Misisi, Zambia.

c Subjects in our USA control cohort were previously enrolled as a branch of SEEM study at Cincinnati Children’s Medical Center, Cincinnati, OH, USA.

**Table 2:** Biomarkers, Stool TaqMan Array Card (TAC) assay, and EEDBI scoring data from the Pakistan EED cohort

Biomarkers at 9 months (units)	Number of subjects sampled (n)	Measured values median (IQR)
C-reactive protein (mg/dL)	48	0.2 (0.1, 0.6)
Urine creatine (umol/L)	52	122.68 (77.82, 181.94)
Ferritin (ng/mL)	48	21.50 (9.50, 54.00)
Hemoglobin (g/L)	49	10.2 (9.0, 11.3)
Prealbumin (mg/dL)	30	13.7 (11.8, 16.1)
Alpha-1-acid glycoprotein (mg/dL)	48	111.0 (85.5, 139.5)
Urine claudin15 (ng/mL)	52	1.3, (0.7, 2.4)
Glucagon-like pepide 2 (pg/mL)	31	1,101.1 (754.7, 1411.6)
Leptin (pg/mL)	31	180.81 (94.06, 271.91)
Stool myeloperoxidase (ng/mL)	51	3,050 (979.5, 6475)
Tumor necrosis factor alpha (pg/mL)	50	57.175 (35.5,113.03)
Interferon gamma (pg/mL)	50	7.995 (0.84,39.72)
Rota-IgA level (100 μL)	48	48.6 (8.7, 160.5)
Serum sodium (mEq/L)	51	137.0 (135.0, 138.0)
Serum potassium (mEq/L)	51	4.5 (4.2, 4.7)
Serum bicarbonate (mEq/L)	51	19.0 (17.1, 20.7)
Anti-tissue transglutaminase IgA	42	1.8 (1.2, 5.0)
Red blood cell count (x10^12^/L)	52	4.7 (4.4, 5.3)
Mean corpuscular volume (fL)	52	68.5 (64.0, 73.1)
Neutrophil count (x10^9^/L)	52	28.6 (21.3, 35.6)
Eosinophil count (x10^9^/L)	52	5.6 (2.4, 9.2)
Platelet count(x10^9^/L)	52	504.5 (408.8, 593.0)
International Normalized Ratio	52	1.0 (0.9, 1.0)

Stool TaqMan (TAC) Analysis between 3-6 months of age	Number of patients negative (n)	Number of patients positive (n)
Astrovirus	3	5
Campylobacter spp.	3	34
CTX-*M*-15–producing enteroaggregative *E. coli*	20	16
aaiC toxin-producing enteroaggregative *E. coli*	3	17
aatA toxin-producing enteroaggregative *E. coli*	3	34
Norovirus G-1	3	4
Norovirus G-2	5	13
shigatoxigenic *E. coli*	4	19

Stool TaqMan (TAC) Analysis at 9 months of age	Number of patients negative (n)	Number of patients positive (n)
Campylobacter spp.	8	23
Cryptosporidium	3	5
CTX-*M*-15–producing enteroaggregative *E. coli*	16	12
aatA toxin-producing enteroaggregative *E. coli*	3	28
enteropathogenic *E. coli*	3	20
Norovirus G-2	4	8
Rotavirus	5	4

Environmental Enteric Dysfunction Biopsy Initiative (EEDBI) Histology Scoring^a^	Median score for *n* = 52 patients from Pakistan EED cohort median (IQR)
Criterion (minimum-maximum score)	**Median Score** (IQR)
Acute (neutrophilic) inflammation (0–3)	0 (0, 0)
Eosinophilic infiltration (0–3)	0 (0, 0.1)
Chronic inflammation in the lamina propria (0–3)	1.3 (1.2, 1.9)
Intraepithelial lymphocytes (0–4)	2.6 (2.3, 2.8)
Villous architecture (0–4)	1.5 (1.1, 1.7)
Intramucosal Brunner glands (0–3)	0.7 (0.3, 0.8)
Foveolar cell metaplasia (0–3)	0.0 (0.0, 0.0)
Goblet cell density (0–4)	1.1 (0.7, 1.3)
Paneth cell density (0–3)	1.0 (0.7, 1.1)
Enterocyte injury (0–3)	0.3 (0.2, 0.7)
Epithelial detachment (0–4)	0.6 (0.4, 0.7)
Total histologic score (0–37)	9.3 (7.1, 10.0)

E. coli, Escherichia coli; EED, environmental enteric dysfunction; *ssp*., subspecies; EEDBI, environmental enteric dysfunction biopsy initiative.

a The EEDBI consortium developed a scoring system to assess intestinal damage in EED. Lower scores signified less degree of inflammation and intestinal architectural distortion, while higher scores represented the reverse. The detailed scoring system can be found in Table S10.^7^

As part of the Biomarkers of Environmental Enteropathy in Children (BEECH) in Misisi, Zambia, the caregivers of *n* = 119 with height-for-age (HAZ) consistently below −2 over 4–6 months of observation after nutritional intervention consented to endoscopy. A subgroup of *n* = 30 patients (Table 1) with hematoxylin & eosin-stained slides available was also sampled for RNA sequencing. Data from this subgroup was used for this analysis. These patients had a median age of 1.1 years (IQR: 0.4,1.3 years) and a median HAZ score of −3.3 (IQR: −3.9, −2.8) (Table 1).

### Machine learning model shows high performance in classifying disease versus controls

**Figure 1:**
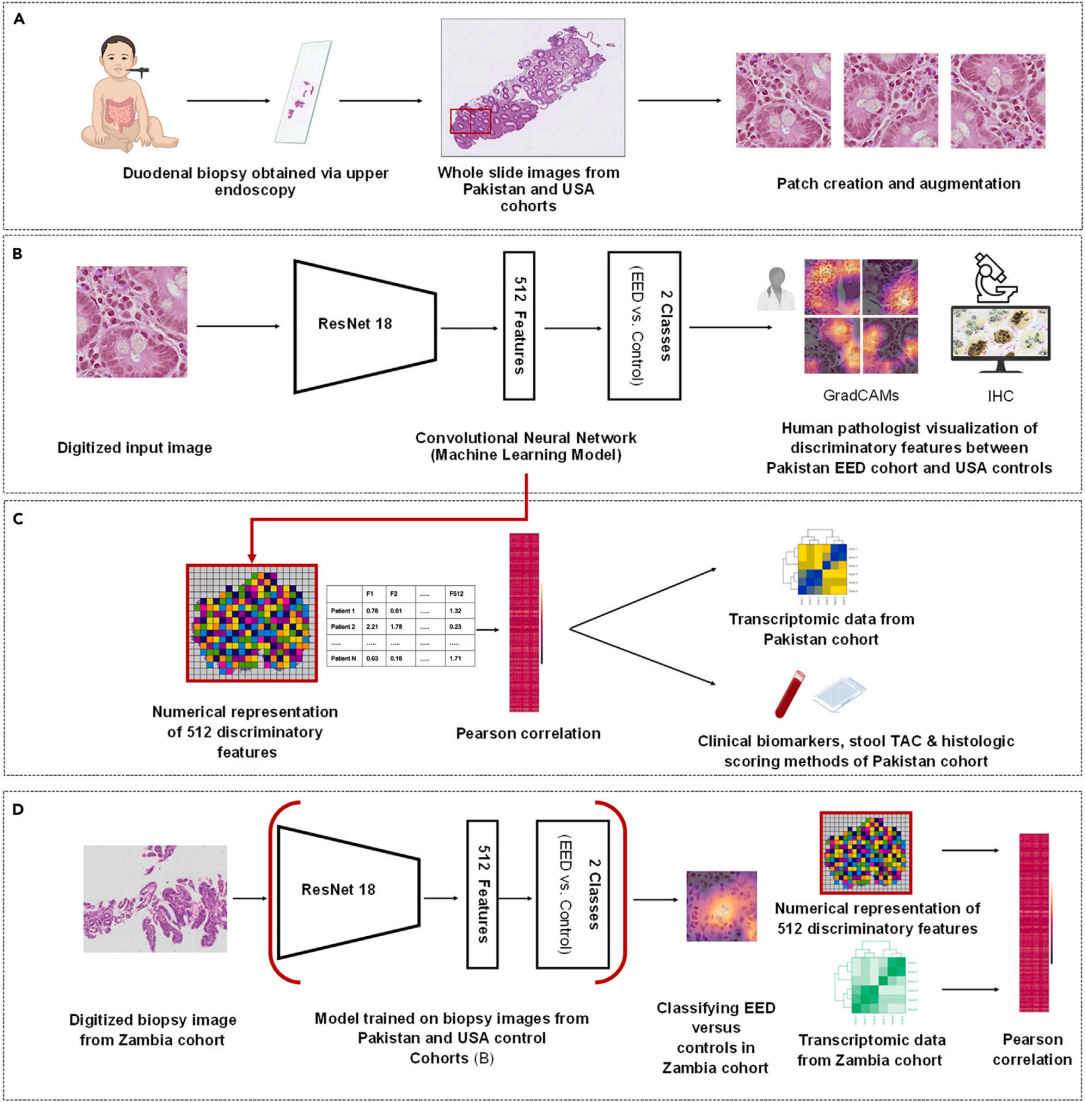
Overview of genotype-phenotype analyses (A) Duodenal tissue biopsies from subjects in Pakistan with EED (*n* = 52) and controls (*n* = 25) were stained with hematoxylin and eosin and digitized as WSIs (*n* = 168 and 56, respectively). WSIs were cropped into patches of 512x512 pixels. Patches were augmented through rotation and stain normalization techniques to ensure these patches were well-distributed on a color histogram spectrum and had multiple morphological patterns. Parallelly, an EED-specific immunohistochemistry panel was applied on unstained duodenal biopsy sections from a subset of the Pakistan cohort (*n* = 21) and USA control (*n* = 20). (B) Patches were input into a ResNet18, a convolutional neural network, to create a classification model. Discriminatory tissue features from the classification model were represented as mathematic vectors. A trained pathologist visualized discriminatory features using GradCAMs to validate the GradCAM-defined features of EED. (C) Histologic features of interest were represented numerically and correlated with transcriptomic data, clinical biomarkers, fecal microbiota (as measured by stool TAC), and histology scores via previously established metrics. Genes of interest underwent functional enrichment analysis as well as comparisons to metabolic network modeling and lipidomic pathway analysis for congruence. (D) Duodenal biopsy WSIs (*n* = 60) from *n* = 30 subjects with EED from Zambia were input into the pre-trained model from Figure 2B to validate model performance. Transcriptomic data from the Zambia cohort was correlated with machine learning-derived feature representations, and the overlap between the datasets from Pakistan and Zambia was analyzed. EED = environmental enteric dysfunction; GradCAMs = Gradient-weighted Class Activation Mappings; H&E: hematoxylin and eosin; IHC = immunohistochemistry; TAC = Taqman Array Card; WSI = whole slide images.

**Figure 2:**
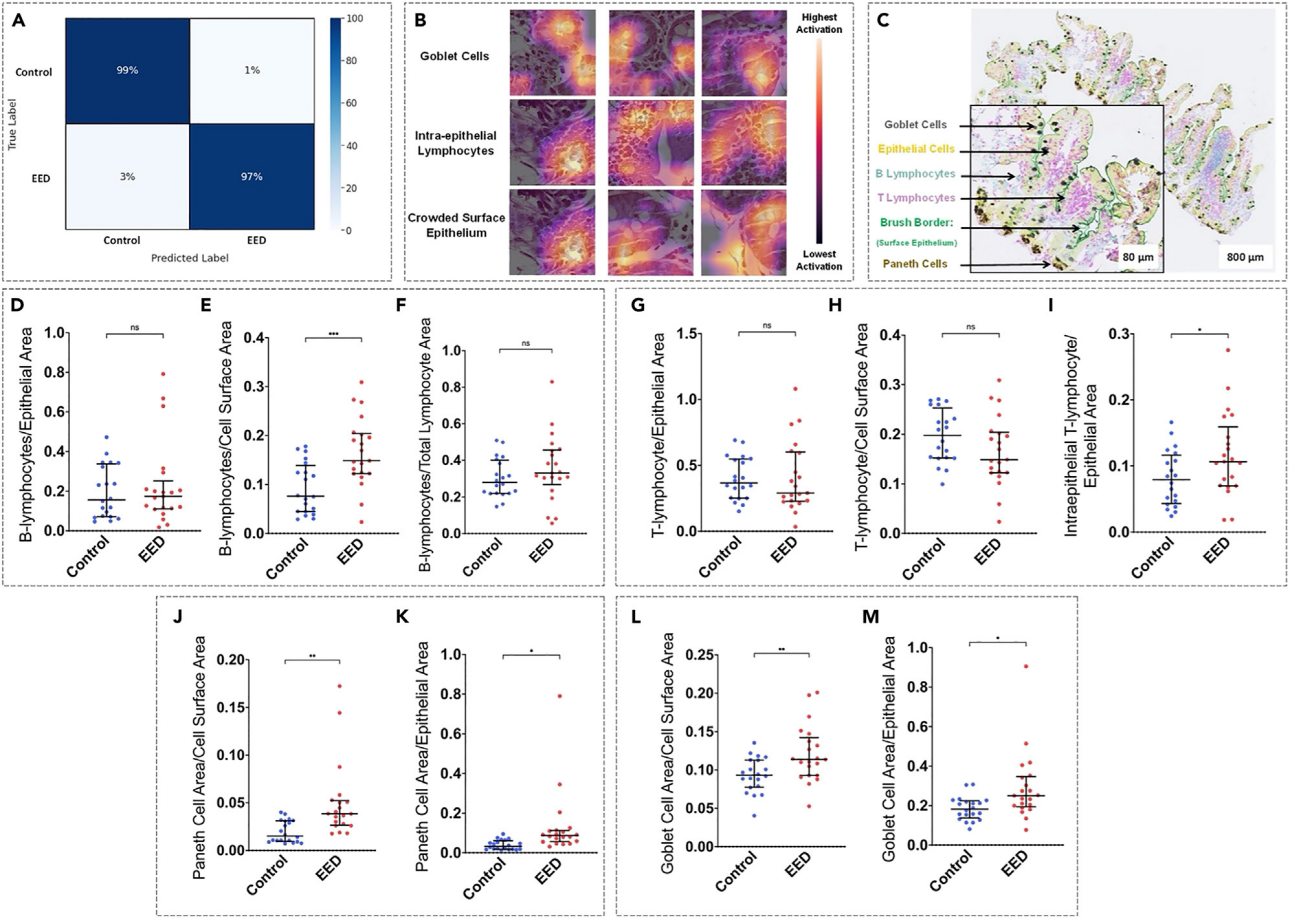
Machine-learning-based classification of EED versus controls validated using immunohistochemistry (A) Patch-level confusion matrix showing the classification of controls versus patients with EED from the Pakistan cohort. The model classified controls correctly in 99% of the images and EED in 97% of the images. (B) Gradient-weighted Class Activation Mapping (GradCAM) review of Pakistan EED images revealed that goblet cells, intraepithelial lymphocytes, and crowded surface epithelium were features of interest in our model. (C) Immunohistochemical staining of the duodenal section using anti-defensin-alpha 5, anti-CD3, anti-cytokeratin, anti-CD 19, anti-sucrase isomaltase, and anti-mucin 2. The scale bar = 800 μm in the non-magnified image and 80 μm in the magnified inlay. (D–M) Box and whisker plots showing median, upper and lower quartiles, and minimum and maximum values. Immunohistochemistry of biopsy slides from patients with EED showed increased intraepithelial lymphocytes, with B-lymphocytes significantly different across the whole tissue surface area, and T-lymphocytes were significantly different in the epithelium. Further, increased Paneth cell area and increased goblet cell area were observable in patients with EED versus controls. ns = not significant, asterixis denote significance: ∗*p* ≤ 0.05, ∗∗*p* ≤ 0.05, ∗∗∗*p* ≤ 0.005. EED = environmental enteric dysfunction.

First, we developed a machine learning-based image model to classify biopsy patches as coming from a subject with EED or control (Figure 1A). The Pakistan EED cohort comprised patients with stunting refractory to nutritional intervention and yielded 168 whole slide images (WSIs) split into 47,402 patches. The 56 WSIs from the control cohort from the USA were subsequently cropped into 30,019 patches. All patches measured 512 x 512 pixels. Next, patches were selected from random splits of the training set and used to train a convolutional neural network-based machine learning classification model (ResNet18). All patches from control subjects were assumed to have no disease, and all patches from EED subjects were assumed to have disease.^21^ 80% of the patches were used to train the model, and 20% to test the model on previously unseen images. This model exhibited an accuracy of 99% at the patch level for classifying EED versus controls in the test dataset (Figure 2A).

To illuminate the "black box” or the decision-making process of the machine learning model, we used gradient-weighted class activation mapping (GradCAMs, Figure 1B). GradCAM saliency maps enabled us to visualize the relative contribution of each of the different regions of the tissue patch toward predicting the probability of that patch belonging to either the disease or control group. Upon the qualitative assessment of these GradCAMs by two trained pathologists at the University of Virginia (UVA), lymphocytes in the surface epithelium and lamina propria, areas containing goblet cells, and areas with crowded surface epithelial cells contributed to a higher prediction probability that a patch came from a subject with EED versus controls (Figure 2B).

### Immunohistochemistry validates gradient-weighted class activation mapping findings of environmental enteric dysfunction features

As a quantitative assessment of the tissue findings of the machine learning model, immunohistochemistry (IHC) was performed on a randomly sampled subset of the Pakistan cohort (*n* = 21) and USA control (*n* = 20) subjects. Upon quantitative analysis, B-lymphocytes and T-lymphocytes were significantly increased across the tissue surface area (*p* ≤ 0.05) when comparing EED tissue against controls (Figures 2D–1I). Although the trained machine-learning model did not highlight Paneth cells to be a region of interest, we noted a statistically significant difference in Paneth cell area in both the epithelial (*p* ≤ 0.05) and total tissue surface area (*p* ≤ 0.01) between subjects with EED and controls (Figures 2J and 2K). In addition, the goblet cell area (Figures 2L–2M) was significantly increased in both the epithelial and total tissue surface area (*p* ≤ 0.05).

### Correlations between RNA sequencing data and 512 ML-derived tissue feature representations

Having developed a model for identifying signature tissue features of EED, we next analyzed potential transcriptome-phenotype interactions by studying the correlations between cellular morphology and gene expression levels (Figures 1C and 1D). For every subject, 512 mathematical vector representations of tissue features were inferred by the ML-based classifier as discriminatory between EED and non-diseased tissue. These 512 features represent the visual structures in a whole slide image of a subject’s biopsy. Reducing a biopsy to these mathematical tissue feature representations is analogous to giving a biopsy a detailed fingerprint unique to each subject.

**Figure 3:**
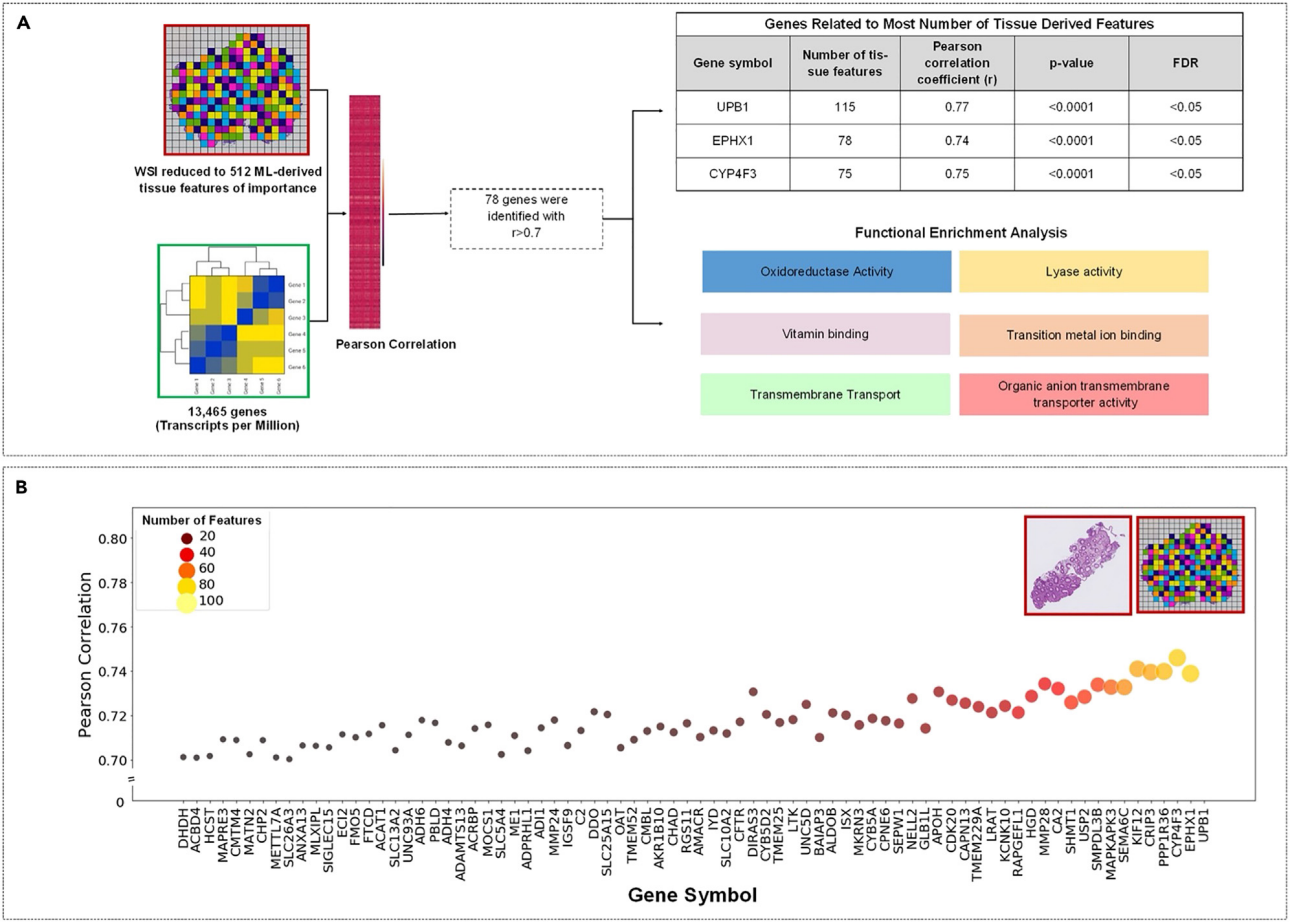
Genotype-phenotype relationship analysis of the Pakistan cohort (A) Machine learning-derived discriminatory tissue feature representations from the classification model were correlated with genomics data using the Pearson correlation coefficient. 78 gene groups most heavily correlated with tissue features (r > 0.7) were analyzed using functional enrichment analysis using ToppGene. Gene groups involved in oxidation and reduction, lyases, vitamin binding genes, transition metal ion binding genes, and genes involved in transmembrane transport were found to correlate strongly with biopsy features. (B) Genes encoding beta-ureidopropionase (UPB1, epoxide hydrolase 1 (EPHX1), and cytochrome p450 4F3 (CYP4F3) were correlated with the highest number of features. *p* < 0.05 was considered statistically significant. ML = machine learning; FDR = false discovery rate using the Benjamini-Hochberg method r = Pearson’s correlation coefficient; WSI = whole slide image.

To correlate biopsy features with gene signatures, we used publicly available bulk RNA sequencing data to obtain gene expression data from patients with EED from the Pakistan cohort. The RNA sequencing data included quantitative expression levels for 13,464 protein-coding genes. We performed pairwise gene-feature correlations for all 13,464 genes and 512 mathematical feature representations. Single genes that correlated to any machine-learning-derived features with a Pearson coefficient greater than 0.7 were extracted. From this analysis, we found 78 genes to be highly correlated (r > 0.7) with 158 of the 512 ML-identified features (Figure 3A).

To determine the functional relevance of these genes, we next used Toppgene^22^ to perform functional enrichment analysis and cluster the genes into distinct molecular groups. Of the 78 genes highly correlated with tissue features, 13 were related to oxidoreductase activity—the most common among the gene set. The remaining genes were found to be related to transition metal ion binding, lyase activity, vitamin binding, and biological processes involved in the transport of small molecules, carboxylic acids, organic acids, and amino acids (Figure 3A). Using the Human Protein Atlas, all 78 genes of interest were studied individually to assess their roles in known cellular processes and whether their disruption could lead to the pathologic changes seen in EED. Genes involved in oxidation and reduction, such as ADH4, ADH6, DHDH, CYP4F3, and CYB5A (enrichment *p*-value <0.005, FDR <0.05), were found to be enriched, as well as various lyases (SHMT1, FTCD, ALDOB, ME1, MOCS1, CA2, enrichment *p* value < 0.005, q-value <0.5), vitamin binding genes, and transition metal ion binding genes, as well as genes involved in transmembrane transport, all of which were found to correlate strongly with biopsy features (r > 0.7). These genes had enrichment *p*-values well below 0.005 and FDR q values < 0.05 using the Bejamini-Hochberg correction (Figure 3A).

Lastly, we sought to determine the number of single tissue feature representations correlated with an individual gene. When all 78 genes were visualized as a function of the number of tissue feature representations they correlated to, the genes beta-ureidopropionase 1 (UPB1), epoxide hydrolase 1 (EPHX1), and cytochrome P450 family 4 subfamily F member 3 (CYP4F3) were determined to correlate to the most features (Figure 3B). Some genes, including dihydrodiol dehydrogenase (DHDH), acyl-CoA binding domain containing 4 (ADBD4), hematopoietic cell signal transducer (HSCT), and multiples genes in the solute-carrier gene (SLC) superfamily including SLC26A3, SLC13A2, SLC10A2, SLC25A15, and SLC5A4, were highly correlated to a small number of mathematical tissue feature representations (Figure 3B).

### Machine-learning-derived tissue feature representations correlate with biomarkers, stool TaqMan array card, and histologic scoring

Next, we sought to investigate the relationship between all the 512 machine-learning-derived tissue feature representations and clinical biomarkers, fecal microbial markers, and histologic scoring indices. To this end, we ran linear correlation analyses between biomarkers, stool TAC data, and EEDBI histology scores with the tissue feature representations. Of the biomarkers analyzed, we found that HAZ and C-reactive protein highly correlated with tissue features, with correlation coefficients greater than 0.6. In addition, between 3 and 6 months, stool TAC samples were positive for enteroaggregative *Escherichia coli, Campylobacter,* and *Cryptosporidium*. *Campylobacter,* Astrovirus, CTX-*M*-15–producing enteroaggregative *Escherichia coli* were positive at 9 months. These stool TAC markers significantly correlated (r < 0.6) with between 50 and 91 features, as detailed in the supplemental information.

Lastly, all 512 machine-learning-derived tissue feature representations were linearly correlated with the (EEDBI) Consortium histology scoring system for *n* = 52 patients with EED. The EEDBI score assesses intestinal damage associated with EED on a numerical scale, with 0 indicating minimal or no damage, while higher values indicate greater damage. These scores are reported in Table 2. Paneth cell density (r = 0.4), villus architecture (r = 0.4), and goblet cell density (r = 0.4) correlated with the greatest number of features, albeit with weakly positive correlation coefficients ranging from 0.35 to 0.40 (Figure S1).

### Validation of machine learning classification model in an external dataset

To ensure our model was not overtrained on site-specific features, we next sought to validate our model on an external dataset. To this end, we evaluated the performance of the machine learning model on 3484 patches (60 digitized biopsies) from 30 children with EED in Zambia (Table 1). When these images were input into our model established above, we obtained a classification accuracy of 88%. Next, we ran our gene correlation pipeline on the RNA sequencing data from the Zambia dataset to correlate the 34,660 identified genes with the 512 machine learning-derived tissue features. 6,189 genes were found to significantly correlate (r > 0.7) with tissue feature representations. Remarkably, 46 genes highly correlated with tissue features of EED in both the Pakistan and Zambia datasets, including CYP4F3, SLC25A15, SLC10A2, SLC26A3, DHDH, and EPHX1.

### Identification of reactions with altered flux in the Pakistan cohort using metabolic network modeling

**Figure 4:**
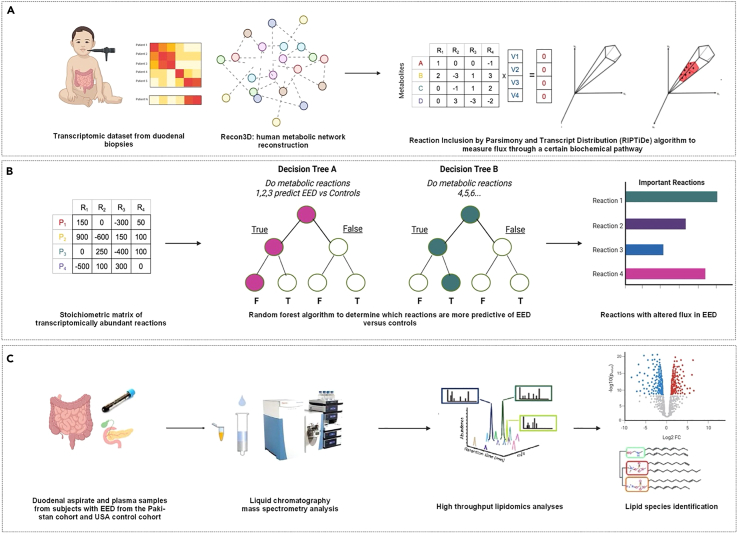
Overview of contextualized metabolic network modeling methods and untargeted mass spectrometry-based metabolomics (A) RNA transcriptomic data from the duodenal biopsies of children with EED from Pakistan and Zambia and controls from the USA were overlaid onto Recon3D, a large, publicly available human metabolic network reconstruction, which contains detailed information on human genes, the proteins they encode, and the metabolic reactions they catalyze. Reactions present in either the transcriptomic datasets from EED or the controls underwent RIPTIDe, a subtype of flux balance analysis. (B) A random forest classifier was used to classify between EED and controls by identifying reactions that were more predictive of either state to generate a list of reactions with altered flux in EED and controls. These reactions were then grouped into their broad biochemical families. (C) Liquid chromatography with high resolution mass spectrometry analysis was performed on both plasma and duodenal aspirate to reveal alterations in lipidome between patients with EED and controls. EED = environmental enteric dysfunction; RIPTIDe = Reaction Inclusion by Parsimony and Transcript Distribution.

Since we found several genes that significantly correlated to features of EED, we hypothesized that these genetic and tissue features might result in differences in the metabolome that could serve as peripheral biomarkers or metabolic signatures of EED. To test this hypothesis, we used a computational metabolic modeling approach to generate metabolic profiles of the disease (Figures 4A and 4B). This approach overlays transcriptomic data from EED or control datasets onto a genome-scale human metabolic reconstruction (Recon3D), to generate a duodenum-specific metabolic model of EED. We then applied the RIPTiDe algorithm to transcriptomic data from subjects with EED enrolled in the SEEM study (*n* = 52) and controls (*n* = 25). This metabolic modeling revealed that the activity of approximately 290 reactions coupled to multiple genes varied between the control cohort and subjects with EED. Next, we sought to test whether this modeling could be used to classify disease. Remarkably, a random forest classifier achieved an accuracy of 64% after 100 train-test splits when determining whether a metabolic profile came from a subject with EED or the control group from the SEEM study. Reactions with an altered rate of flow down a metabolic reaction (also known as metabolic flux) that predicted disease in multiple runs of the model were explored using the Recon3D reaction database.

**Figure 5:**
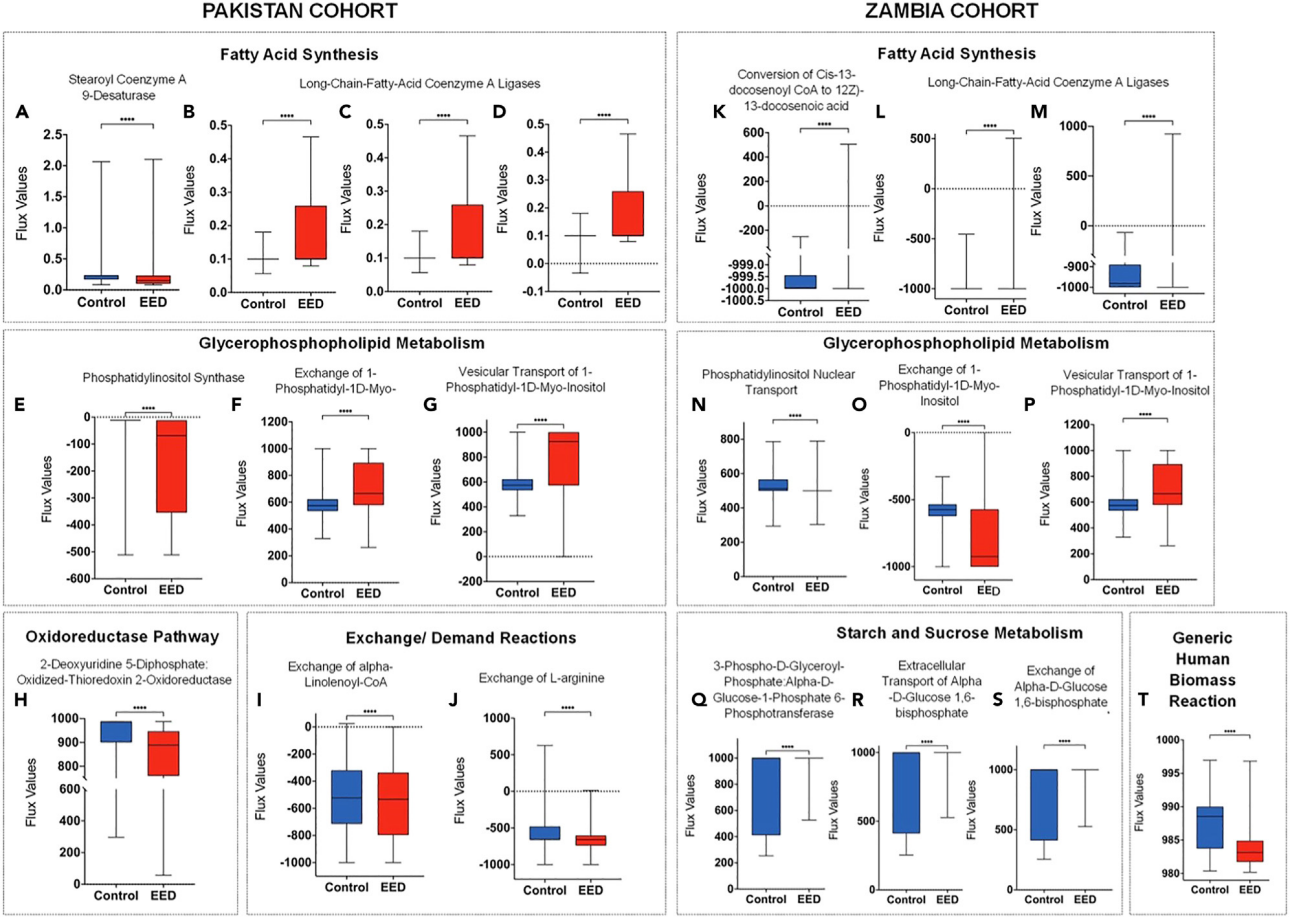
Reactions with altered flux in EED versus controls from the USA Box and whisker plots showing median, upper, and lower quartiles, and minimum and maximum values. Our Flux Balance Analysis/Random Forest framework was applied to RNA sequencing data from the Pakistan EED cohort (A-J) or Zambia EED cohort (K-T) and compared to controls from the USA. The most altered metabolic reactions between the EED and control groups were categorized into "families" based on the broad biochemical processes they catalyze. In all graphs, the x axis describes the altered reaction between the controls (white) and EED states (gray). The y axis shows the flux values generated by RIPTiDe after analyzing the flow of metabolites through a duodenal-specific metabolic network reconstruction. The scale of flux values (y axis) varies with the reactions as the efficiency of different metabolic pathways in generating biomass varies in a given biological system. A Mann-Whitney U test was used to compare reactions that varied between patients with EED and controls. The scale of flux values (y axis) varies with the reactions as the efficiency of different metabolic pathways in generating biomass varies in each biological system. ∗∗∗∗*p* < 0.0001. EED = environmental enteric dysfunction.

The 10 most commonly ranked reactions which differentiated EED and control include stearoyl coenzyme A 9-desaturase (HMR_2296), long-chain-fatty-acid coenzyme A ligases (HMR_0279, HMR_0241, and HMR_0385), phosphatidylinositol synthase (CDIPTr), vesicular transport of 1-phosphatidyl-1D-myo-inositol (PAIL_hs_t1e), exchange of 1-phosphatidyl-1D-myo-inositol (EX_pail_hs[e]), 2-deoxyuridine 5-diphosphate: oxidized-thioredoxin 2-oxidoreductase (r1431) exchange of alpha-linolenoyl-CoA (sink_lnlncacoa[c]), and the exchange of L-arginine (sink_arg_L[c]) (Figures 5A–5J).

These reactions were then grouped by biochemical systems they have been studied to be involved in using the Recon3D database.^23^ The reactions found to be significantly altered in the Pakistan EED dataset belonged to the fatty acid synthesis (Figures 5A–5D), glycerophospholipid metabolism (Figures 5E–5G), the oxidoreductase pathway (Figure 5H), and exchange/demand reactions, or pseudo reactions, representing a summation of biological functions rather than specific enzyme-catalyzed metabolic pathways (Figures 5I and 5J).^24^

### Reactions with altered flux in the Zambia cohort

Subsequently, we sought to determine whether the Zambia EED cohort also displayed a specific metabolic signature. Using the metabolic modeling methods outlined above, we found 145 reactions to be altered in subjects with EED from Zambia compared to controls, with a random forest model accuracy of 97% in classifying an EED versus control. We then identified discriminatory reactions and grouped them into their broad biochemical families (Figures 5K–5T). Similar to the Pakistan model, reactions involved in fatty acid synthesis (Figures 5K–5M) and glycerophosphopholipid metabolism (Figures 5N–5P) also had an altered rate of flux in EED, while reactions involved in starch and sucrose metabolism (Figures 5Q–5S) and a generic human biomass reaction (Figure 5T).

### Validation of in silico metabolic modeling approach using untargeted lipidomics

**Figure 6:**
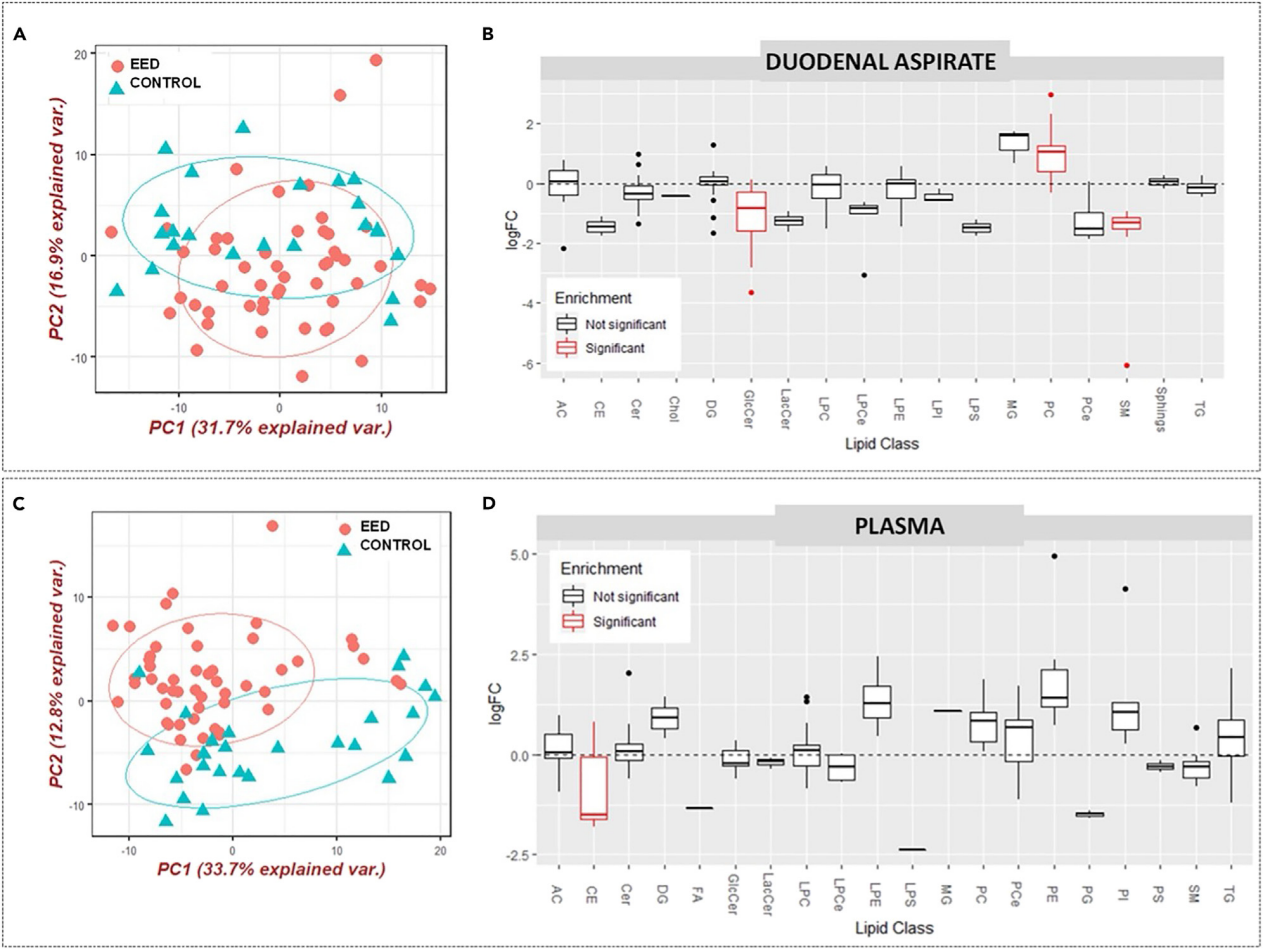
Untargeted lipidomics analyses of EED cohort from Pakistan and controls from the USA (A and B) Lipidomic analysis of duodenal aspirate. (C and D) Lipidomic analysis of plasma. (A and C) Principal component analysis of EED versus control groups from the duodenal aspirate and plasma, respectively. Ion features from positive ES mode were used in the analysis. Explained variance is a statistical measure of the amount of variation in a dataset that can be ascribed to each of the principal components obtained by the principal component analysis technique. (B) Lipid set enrichment analysis (LSEA) showed sphingomyelin and glucosylceramide classes significantly downregulated and phosphatidylcholines were upregulated in the duodenal aspirate. (D) LSEA showed cholesterol esters were significantly down-regulated in the plasma. After lipids are ranked by their fold changes, enrichment scores, and significance are calculated for each lipid set using an efficient permutation algorithm^85^. The x axis shows the lipid classes (detailed nomenclature found in Tables S4–S9). The y axis shows the logarithmic transformation of the fold change (logFC). PC = principal component; EED = environmental enteric dysfunction.

The collection of paired samples of duodenal aspirate and plasma at endoscopy in the previously conducted SEEM study permitted us to profile both plasma lipids and intraluminal duodenal lipids in subjects with EED (Figure 4C). Principal component analyses showed distinct variance in patients with EED and controls (Figures 6A and 6C)

Using the ultra-performance liquid chromatography high-resolution mass spectrometry, we found that members of the glycerophospholipid family, including phosphatidylcholine (PC), lysophosphatidylcholine (LPC) and ether-linked LPCs were increased in the duodenal lipidome of children with EED from Pakistan, while ether-linked PCs were decreased. In the plasma, we observed hydroxylated FAs to be significantly lower in the plasma of children with EED (Figures 6B and 4D), in contrast with significantly high levels observed in the duodenal aspirate of this group.

Additionally, triglycerides (TGs) with very long and polyunsaturated fatty acyl chains were decreased in the duodenal lipidome of children with EED, while TGs with saturated and monounsaturated fatty acyl chains increased compared to the control group. The duodenal aspirate of children with the disease also exhibited a relative sphingomyelin deficiency. Lastly, glycine-conjugated bile acids such as glycocholic acid (GCA) and glycochendeoxycholic (GCDCA) were significantly increased in the plasma of children with EED but decreased in the duodenal aspirate. Other significantly altered lipids detected in this analysis include cholesteryl esters, glucosylceramides, phosphatidylethanolamine (PE), and phosphatidylinositol (PI) (Figures 6B and 6D).

## Discussion

In this article, we have extracted tissue feature representations from a machine-learning-based image classifier and correlated them with gene expression data and clinical biomarkers to examine the pathophysiology of environmental enteric dysfunction. First, we trained our ML-based model to classify between EED versus control patients from Pakistan. We validated this model using a geographically distinct external dataset from Zambia. We then extracted tissue feature representations from our classification model for EED in Pakistan and correlated it with transcriptomic data, clinical and stool biomarkers, and a histologic scoring index from the same dataset. Additionally, we explored the relationship between transcriptomics and intestinal function to establish a metabolic signature of EED in Pakistan, which was validated using untargeted mass spectrometry-based lipidomics. These analyses together elucidate a multi-omic signature of EED.

The current mainstay for the diagnosis of EED is tissue biopsy and histologic evaluation by a pathologist.^10^ Previous studies have recently sought to establish semi-quantitative diagnostic criteria that accounted for the villus atrophy and intraepithelial lymphocyte infiltration observed in EED.^8^ In tandem with gastroenterologists and pathologists, the EED Biopsy Initiative Consortium developed a scoring system for evaluating EED from pediatric biopsies. This study found goblet and Paneth cell depletion and intraepithelial lymphocyte infiltration hallmarks of EED.^6^^,^^7^ GradCAM analysis of our model revealed goblet cells and lymphocytes to be important in classifying a biopsy as disease versus control. Notably, goblet cells have previously been implicated in adaptive intestinal epithelial response in EED to improve barrier function. Further, T-lymphocytes have multifaceted immunologic roles in the intestine, including protection from pathogens while tolerating commensal microbiota species.^25^ T lymphocytic infiltration is a hallmark of inflammatory enteropathies, including celiac disease and inflammatory bowel disease.^26^^,^^27^ Another group from Zambia studying EED found that goblet cells, which play a role in maintaining the epithelial barrier, are depleted in cohorts of Zambian children with EED.^28^ GradCAM review of the outputs of our machine learning model shows that this model may also utilize these histologic features to discriminate between disease and controls, underscoring the role of upregulated innate host responses in disease pathogenesis.

Used ML-based image analysis methods, we were able to highly correlate mathematical tissue feature representations with 78 genes (r > 0.7). Interestingly, we observed several genes highly correlated to many tissue features, while others correlated with as few as a singular feature. Of those genes that correlated to over 100 features, beta-ureidopropionase (UPB1) catalyzes the final step of pyrimidine metabolism by converting N-carbamyl-beta-alanine to beta-alanine, a key mediator of immune homeostasis.^29^^,^^30^ A previous study of the Pakistan EED cohort as part of SEEM found UPB1 was downregulated in patients with EED compared to controls.^20^ Transcriptomic studies of celiac disease have also found UPB1 to be downregulated in celiac disease, an inflammatory enteropathy with shared histologic features with EED.^31^

In one study of the Pakistan cohort, targeted metabolomics using tandem mass spectrometry revealed that a high proportion of undernourished children had persistently elevated total sulfated bile acid concentrations, a pattern consistent with hepatic injury.^32^ Notably, we also found bile acids to be increased in the plasma but decreased on metabolomic analysis of the duodenal aspirate of children with EED from Pakistan, consistent with previous analyses.^32^The solute carrier family 10A2 (SLC10A2) gene, which showed strong correlations with approximately 20 tissue feature representations, encodes for the apical sodium-dependent bile acid transporter (ASBT) known to mediate bile acid reabsorption in the ileum and jejunum.^33^^,^^34^ This gene was found to be downregulated through differential expression analysis of the same cohort as well as a disease comparison group with celiac disease.^20^ Changes in the expression of ASBT have been linked to bile acid malabsorption in inflammatory bowel disease.^35^^,^^36^ Further, studies have shown that bile acids in the colon may induce the secretion of water and electrolytes from the colon wall, leading to diarrhea.^37^ Thus, ABST is increasingly becoming a target for pharmaceutical interventions in bile acid-related disorders and may be an avenue for EED therapy.

On flux balance analysis, we found the enzyme stearoyl coenzyme-A 9-desaturase (SCD) to have altered flux in EED. SCD catalyzes the rate-limiting step in the intracellular synthesis of the monounsaturated fatty acids palmitoleate and oleate from the saturated fatty acids palmitate and stearate.^38^^,^^39^ Monounsaturated fatty acids (MUFAs) such as palmitoleate and oleate are the preferred substrates in triglyceride synthesis, phospholipids, and cholesterol esters.^39^^,^^40^^,^^41^ Each lipid class has distinct functions; the upstream perturbation of MUFA synthesis may lead to a spectrum of disturbances in both metabolism and inflammation. SCD is ubiquitously expressed in humans and is highly expressed in tissues with high metabolic activity, such as the liver and adipose tissue.^40^ Mouse models of SCD have shown that high expression levels of SCD are correlated with metabolic diseases, including obesity and insulin resistance. Complete loss of SCD expression has been implicated in a steatohepatitis-like pattern of liver injury, suggesting congruence between this finding, our genotype-phenotype correlation analyses, and previously reported liver involvement in EED.^42^^,^^43^

Another gene highly correlated with tissue features is CYP4F3, a member of the cytochrome P450 superfamily of enzymes involved in the inactivation and degradation of leukotriene B4 (LTB4), a potent inflammatory mediator.^44^ Studies of inflammatory bowel disease show that polymorphisms in this gene may perpetuate intestinal inflammation due to the persistence of LTB4 in the intestinal tissue.^45^ Parallelly, on flux balance analysis, we observed alterations in long-chain fatty acid-CoA ligases, also known as acyl-coenzyme A synthetases long-chain isoform (ACSL), a family of enzymes involved in activating long-chain free fatty acids as the first step for usability by tissues.^46^ One member of this family, ACSL4, is hypothesized to catalyze several polyunsaturated fatty acids (PUFAs) preferentially, one notable substrate being arachidonic acid, which is then metabolized into multiple eicosanoid mediators of inflammation, including LTB4, a finding in line with our genotype-phenotype correlation analysis.^47^^,^^48^

This article illuminates the functional relationships between genotypes and metabolism through contextualized metabolic network modeling and lipidomics that are generalizable to any enteropathy. This presents an exciting new avenue to study genotype-phenotype relationships computationally, which can be used either independently or as a tool to guide experimental platforms studying the effects of gene perturbations in the laboratory.^49^ Furthermore, using GradCAMs allows for visualizing features of interest, illuminating the "black box" of AI. The significant correlations between known propagators of chronic inflammation, the hallmark of EED, and tissue changes resulting from inflammation support probing deeper into the genome of patients with EED. Analysis of genomic alterations may give us further insight into the molecular mechanism by which this combination of inflammation, infection, and growth failure affects children with EED and may illuminate novel targets for therapeutic intervention in the complex disease.

### Limitations of the study

One notable limitation of this study is the difference in the study protocols of the Pakistan and Zambia studies of EED, warranting harmony among future protocols investigating the disease. In the Zambia cohort, the post-operative orientation of a slide under a dissecting microscope was not carried out in the SEEM study, presenting a fundamental confounder in the slide preparation process. The slides were digitized at two sites using two different brands of scanners, which may have resulted in site-specific image differences affecting model performance.^50^^,^^51^^,^^52^ The preparation of samples for RNA sequencing also differed between sites, resulting in large differences in gene count data between the two sites. In our metabolic modeling approach, we used Recon3D, a generic metabolic network model not specific to the duodenum. Further, we used an open-medium in silico model of EED for this exploratory analysis, and including medium information may refine this analysis. We did not have plasma or duodenal aspirate lipidomic data from Zambia for comparison. The controls from the USA had a mean age of approximately five years, while the cohorts with EED from Pakistan and Zambia were around one year old at the time of biopsy. Further, the small sample size across all cohorts and the need for age-matched in-country controls from Pakistan and Zambia are also notable limitations.

## STAR★Methods

### Key resources table

**Table:** 

REAGENT or RESOURCE	SOURCE	IDENTIFIER
**Deposited data**		

Transcriptomic data from the Study of Environmental Enteropathy and Malnutrition (Pakistan)	GEO	GSE159495
Transcriptomic data from the Biomarkers of Environmental Enteropathy in Children	GEO	GSE162630

**Software and algorithms**		

Original Code for the Paper	Github	https://github.com/SyedLab-GI/ee-omics/tree/main
Lipid R Package	Bioconductor	Mohamed et al.^53^

### Resource availability

#### Lead contact

Further information and requests for resources should be directed to and will be fulfilled by the lead contact, Dr. Sana Syed (sana.syed@virginia.edu).

#### Materials availability

This study did not generate new unique reagents.

#### Data and code availability

• This paper analyzes existing, publicly available data. Publicly available transcriptomic data from the Pakistan study was accessed using the GEO accession series GSE159495.^20^ Publicly availble RNA transcriptomic data from the BEECH study was accessed using the GEO accession series GSE162630.^11^ These accession numbers for the datasets are listed in the key resources table.
• The original code used in the methods is available on GitHub at the following link: https://github.com/SyedLab-GI/ee-omics/tree/main. The code is publicly available as of the date of publication.
• Any additional information required to reanalyze the data reported in this paper is available from the lead contact upon request.

### Experimental model and study participant details

#### Selection of clinical cohorts

Data from a population in Pakistan was collected through the Study of Environmental Enteropathy and Malnutrition (SEEM), a prospective cohort study conducted at the Aga Khan University (AKU) in Karachi, Pakistan, between 2016 and 2019. Detailed methods for participant selection are detailed in previous studies.^10^ Ethical considerations prohibit the collection of endoscopic samples from study participants with no clinical indications; therefore, pediatric controls were enrolled at the Cincinnati Children’s Hospital Medical Center (CCHMC) in the United States of America. These patients had been referred for EGD by their providers due to nonspecific gastrointestinal complaints and were subsequently found to have no gross or histopathologic abnormalities on EGD and tissue biopsy.

The validation cohort for this study was enrolled as part of the Biomarkers of Environmental Enteropathy in Children (BEECH) study conducted in Misisi, Zambia.^11^ Distinct from the Pakistan cohort, the biopsies from the Zambia cohort underwent an additional step of biopsy orientation under a dissecting microscope before being embedded in paraffin wax.^11^

The Study of Environmental Enteropathy and Malnutrition was approved by the Aga Khan University Ethical Review Committee (ERC: 2446 Ped ERC13) and the IRB approval at the Cincinnati Children’s Medical Center (CCHMC, Study ID 2016–0387). This study is registered with ClinicalTrials.gov, NCT03588013. The Biomarkers of Environmental Enteropathy in Children (BEECH study was approved by the University of Zambia Biomedical and Research Committee (ref. 006-02-16, 31 May 2016).

#### Transcriptomic data accession and curation

Transcripts per million (TPM) normalized transcriptomic data from the Pakistan study was accessed using the GEO accession series GSE159495.^20^ RNA transcriptomic data from the BEECH study was accessed using the GEO accession series GSE162630 and was processed as fragments per kilobase mapped (FPKM) values.^11^ Owing to the large differences in gene count data between the Pakistan and Zambia datasets, we normalized the BEECH gene expression data to TPM using *kallisto* to quantify transcriptomic abundances on the same scale for both datasets and facilitate analogous correlation analyses.^54^

### Method details

#### Biospecimen collection for biomarker analysis and histologic scoring

We utilized a rich archive of clinical metadata for our correlation analysis with dimension features or tissue feature mathematical representations to aid in discovering potential novel biomarkers for EED. Blood, urine, fecal specimens, and anthropometric measurements were collected from the enrolled subjects at six and nine months of age as part of the Pakistan study.^10^^,^^55^ These included growth measurements, biomarker panels for intestinal barrier structure and function, markers of inflammation, cell counts, serum electrolytes, stool TaqMan array card (TAC), and the EEDBI histology scores from the patients enrolled in the study. Detailed methods can be found in previously published analyses of the Pakistan dataset.^56^^,^^57^^,^^58^

H&E-stained biopsy slides were evaluated centrally at AKU by two pathologists familiar with EED histologic features. The images were scored based on the criteria in the Environmental Enteric Dysfunction Biopsy Initiative (EEDBI) Consortium histology scoring system.^7^

#### Biopsy image digitization, pre-processing, and augmentation

Hematoxylin and eosin (H&E) stained biopsy slides from Pakistan and the USA were digitized at high resolutions (averaging 20,000 by 20,000 pixels) at a magnification of 40X using an Olympus VS 120 (Olympus Corporation Inc., Center Valley, PA) and Leica SCN400 bright field scanning microscope (Leica Microsystems CMS GmbH, Germany) in Pakistan and the United States, respectively. H&E-stained biopsies from the Zambia cohort were scanned on an Olympus VS120 slide scanning microscope (Olympus Corporation Inc., Center Valley, Pennsylvania) at 20X magnification.

High-resolution whole slide duodenal biopsy images were split into patches of 512 x 512 pixels to accommodate the computational limitations of our graphics process unit.^59^^,^^60^ There was no tissue overlap in each patch. Patches that contained less than 50% of the tissue were discarded. In this way, the patches are only extracted from regions with high amounts of tissue, thus most likely representative of the disease histopathology.^61^^,^^62^ To account for stain variations resulting from the preparation and scanning of the slides at different sites, we employed stain color normalization using structure-preserving methods to eliminate bias due to color differences in the biopsies from different sites.^63^ This process involved empirically selecting a target patch to normalize the stain color across all patches. Artificial data augmentation techniques, including segmentation, vertical reflection, horizontal reflection, and γ correction of randomly selected patches, were performed. A random subset of artificially augmented patches was reviewed by data scientists and pathologists to ensure these patches were well-distributed on a color histogram spectrum and collectively represented multiple morphological patterns. These data augmentation techniques allowed for adjusting the training data for artifacts, hue, and magnification-based image variations^64^^,^^65^ (Figure 1A).

#### Training a machine-learning-based classification model for image analysis

ResNet18, a widely used deep convolutional neural network (CNN) architecture with 18 layers for image classification, was trained to classify disease versus control patches.^66^ As the biopsy patch moves through each layer, the model learns microscopic tissue features of the image, assigning a numerical value above zero to each, forming a mathematical "picture" or tissue feature map of the image. These numerical feature representations are condensed at each layer, reducing the image from the HxWx3 matrix representation to a two-dimensional final output layer per patch, highlighting the probability of the patch being either from a patient with a disease or a control patch. In addition, the N-dimension (N=512 for ResNet18) pre-final layer feature representation of images extracted from these models contains a morphological scoring of each patch. These scores contain the patterns deemed relevant by machine learning for distinguishing between disease and normal patients. As a first step, biopsies from the Pakistan cohort and controls from the USA were used to train the ResNet18 model, with 80% of images used for training and the remaining 20% to test the model. To test the model in an unbiased fashion, we used patient-level validation to ensure the same patient was not used in both the testing and training phases, and thereby there was no skew in the accuracy metrics. Subsequently, tissue features utilized by the image analysis model for decision-making were visualized using Gradient-weighted Class Activation Mappings (GradCAMs)^67^ and reviewed by two trained pathologists to minimize inter-operator variability (Figure 1B).

#### Immunohistochemistry (IHC) analysis of machine learning-derived findings

To further visualize the features of high importance identified on GradCAMs, triple color immunohistochemistry staining was performed on a subset of Pakistan and control patients at the University of Virginia’s Biorepository and Tissue Research Facility (BTRF). A particular focus was the characterization of immune, goblet, and Paneth cells and their distribution in different tissue parts. In brief, primary antibodies were used to stain goblet cells, B- and T-lymphocytes, Paneth cells, brush border, and surface epithelium, all components of the EEDBI histology-scoring index^7^ (Figure 5). In addition, the antigens CD-19 (teal) and CD-3 (purple) were targeted for B- and T-lymphocytes, respectively. Two gastrointestinal pathologists provided a qualitative assessment of the stained tissue. Subsequently, quantitative IHC stain analysis was done using the Visiopharm Oncotopix software (Visiopharm, Denmark). Differences in the means of the IHC data for patients with EED and controls were assessed using an unpaired t-test using GraphPad Prism version 8.0 for Windows (GraphPad Software, Boston, MA, USA) (Figure 1A).

#### Correlation of ML-derived tissue feature representations and transcriptomic data

The pre-final layer feature representation extracted from the trained machine learning model was used for correlation analysis with the transcriptomics data. However, this required us to aggregate individual patch representation to a WSI representation for obtaining patient-level representation. Mean-pooling and max-pooling are popularly used approaches for aggregating patch-level representations to WSI-level representations, i.e., the diagnosis made for one patient was made after “pooling” all the diagnoses for each patch coming from that patient. We used a probability-weighted average aggregation approach to emphasize the activation of patches with the highest severity of EED using methods previously published in similar genotype-phenotype analyses.^68^^,^^69^ Feature representation of all the patches present in a WSI was extracted from the pre-final layer of our trained deep-learning model. These patch representations were aggregated to a WSI-level representation using the disease prediction probability (EED) as weight. Feature representation of all the patients (EED and controls) was computed and used for Pearson correlation analysis with TPM-normalized gene data (Figure 2C). All statistically significant pairs with a Pearson correlation coefficient (r), also known as the effect size, of greater than 0.7 were extracted and analyzed in three separate ways to contextualize the gene list: 1) Functional enrichment analysis was then performed on the resulting list of highly correlated genes using ToppGene^22^ and visualized using Cytoscape,^70^ 2) analysis on the function of individual genes was carried out using the Human Protein Atlas,^71^ 3) genes were arranged based on the number of machine-learning derived features they correlated (r > 0.7) with and studied for insights into the disease process. The Benjamini-Hochberg procedure was performed to minimize false discovery rates for multiple comparisons, and FDR threshold q-value of <0.05 was used throughout the analysis.^72^

#### Correlation of ML-derived tissue feature with clinical biomarkers

The same tissue feature mathematical representations were extracted from the pre-final layer of the trained model and were used for this analysis. The clinical metadata was divided into clinical biomarkers, EEDBI histology score, and stool TAC data. Pearson correlation analysis was performed for clinical biomarkers and the EEDBI histology scores of our study population, and values with statistically significant relationships (p-value < 0.01) were extracted and used for qualitative analysis. As stool TAC data is categorical, a Mann-Whitney U-test was used for the statistical comparison with the machine-learning-derived tissue features. Again, TAC values with a statistically significant relationship (p-value < 0.05) were extracted and used for qualitative evaluation (Figure 1C).

#### Validation of findings across a geographically distinct dataset from Zambia

Duodenal tissue biopsy images from the Zambia cohort were pre-processed to input into ResNet18 as 512 x 512 pixels patches. Notably, no patches from this cohort were used in the initial training and testing process, and the model was "frozen" after training and testing exclusively on the Pakistan cohort and US controls. In other words, the model did not learn any new features present in the Zambia cohort but rather classified these biopsies based on what it had previously learned when trained on Pakistan and US controls, allowing us to test the generalizability of the model in detecting discriminatory features of EED across geographical sites. Subsequently, GradCAMs were used to identify areas of high importance to the model in its classification decision-making and reviewed by trained pathologists for histopathological logic (Figure 1D).

#### Comparison of gene correlations across different cohorts

RNA transcriptomic data from the Zambia cohort were TPM normalized using kallisto to runn correlation analysis.^54^ Tissue feature mathematical representations were extracted from the machine-learning model’s pre-final layer and aggregated back to WSI-level presentations to obtain patient-level representations. Genes correlated with tissue features with a Pearson correlation coefficient greater than 0.7 were extracted, and the overlap between the Zambia and Pakistan datasets was analyzed.

#### In silico construction of a duodenal-specific metabolic network reconstruction to model EED

RNA sequencing data accessed for gene correlation analysis was overlaid onto Recon3D, a human metabolic reconstruction, to obtain a duodenal-specific model for metabolism. Genes in both the Recon3D network and transcriptomic datasets were retained, while the rest were removed. Retained reactions were specifically linked with transcriptomically-abundant genes in the Pakistan and Zambia datasets. We used the Constraint-Based Reconstruction and Analysis (COBRA) Toolbox to remove reactions determined to be inactive to generate the duodenum-specific metabolic network reconstruction to contextualize our downstream analyses.^73^^,^^74^ Two independent models were constructed for the Zambia and Pakistan datasets for metabolic network modeling (Figure 4A).

#### Identifying reactions with differential utilization of relevant metabolic pathways

We then employed RIPTiDe, a subset of parsimonious Flux Balance Analysis (pFBA), to prune the list of genes obtained from the diseased and control patient cohorts.^75^^,^^76^^,^^77^^,^^78^^,^^79^^,^^80^^,^^81^ Using RIPTiDe, the transcriptomic dataset from Pakistan (SEEM study) was used to create a contextualized metabolic model for each patient based on the parsimonious usage of reactions as defined by their associated transcriptomic abundances. In each patient, multiple possible flux values were generated from the permissible range of flux bounds for all transcriptomically active reactions.^75^^,^^79^ RIPTiDe randomly samples 50 to 500 flux values depending on the permissible flux ranges. For this experiment, the parameters for implementing the RIPTiDe package were kept at their default values, except for the minimum percent of optimal objective value during the flux balance analysis, which we increased from 0.8 to 1 to control the variability of flux ranges. A flux sampling method was used to generate multiple data points for training a machine-learning random forest model using the scikit learn implementation.^82^^,^^83^ We kept the open_exchange parameter in the COBRA optimizer as "false" to allow for the FBA module to automatically determine the maximum and minimum bound of reactions based on transcriptome contextualization and FBA optimization.^81^

#### Extraction of discriminative reactions using machine learning

After using RIPTiDE, the reactions found to have a similar flow between patients with EED, and controls were not used for further investigation, as they were assumed to be reactions essential for healthy tissue function in diseased and non-diseased states.^80^ Reactions found to have altered flux between patients with the disease were used to classify patients as either having EED or as controls. An 80-20 ratio was used to create a training and test dataset for the random forest classification model, and all flux points used to test the model were independent of those used to train it. Extensive cross-validation using multiple splits of train-and-test patient sets was done to demonstrate performance gains and takeaways across the dataset. The top reactions were extracted and aggregated from splits in which the model had the highest classification accuracy (Figure 4B).

#### Grouping reactions to derive functional groups

The top 10 reactions identified by our metabolic modeling pipeline were extracted from the splits with an accuracy greater than 60% and used for functional group analysis. Differences between patients with EED and controls were calculated using the Mann-Whitney U test, and a p-value of <0.5 was considered significant. These top 10 reactions were grouped based on their metabolic subsystems and description of their overall function based on the virtual metabolic human (VMH) reaction database of Recon3D.^23^

#### Untargeted lipidomics analysis on the Pakistan cohort and controls by ultra-performance liquid chromatography coupled to high-resolution mass spectrometry (UHPLC-HRMS)

Paired plasma and duodenal aspirate samples were collected during endoscopy while these children were sedated and fasted. Post-saline lavage duodenal aspirates were collected from the second portion of the duodenum after infusion of 10 to 25 mL saline solution via sterile catheter.^10^ It was not possible to collect samples from all the children at each time point; however, a total sample size was obtained. Both duodenal aspirate and plasma samples from children with EED (Pakistan) and controls were sent and processed at the Clinical Mass Spectrometry Facility at CCHMC before lipidomic analysis (Figure 4C). A p-value <0.05 was considered statistically significant. Lipid set enrichment analysis was conducted by the “Lipidr” R package.^53^ All statistical analysis was conducted in R Language and Environment for Statistical Computing (www.r-project.org).

### Quantification and statistical analysis

The relevant subsections of the methods describe statistical analyses and the software they are performed on related to each experiment, with corresponding results in the relevant results sections. The letter n denotes the number of subjects included in a particular analysis.
