# Supplementary material for: Machine-learning-based integrative –‘omics analyses reveal immunologic and metabolic dysregulation in environmental enteric dysfunction
Source: iScience. 2024 May 17;27(6):110013. doi: 10.1016/j.isci.2024.110013 (PMC11167436; doi:10.1016/j.isci.2024.110013)
Supplement: Document S1. Figure S1 and Tables S1–S9 [file mmc1.pdf]

iScience, Volume *27*

Supplemental information

Machine-learning-based integrative –`omics analyses reveal immunologic and metabolic dysregulation in environmental enteric dysfunction

Fatima Zulqarnain, Xueheng Zhao, Kenneth D.R. Setchell, Yash Sharma, Phillip Fernandes, Sanjana Srivastava, Aman Shrivastava, Lubaina Ehsan, Varun Jain, Shyam Raghavan, Christopher Moskaluk, Yael Haberman, Lee A. Denson, Khyati Mehta, Najeeha T. Iqbal, Najeeb Rahman, Kamran Sadiq, Zubair Ahmad, Romana Idress, Junaid Iqbal, Sheraz Ahmed, Aneeta Hotwani, Fayyaz Umrani, Beatrice Amadi, Paul Kelly, Donald E. Brown, Sean R. Moore, Syed Asad Ali, and Sana Syed

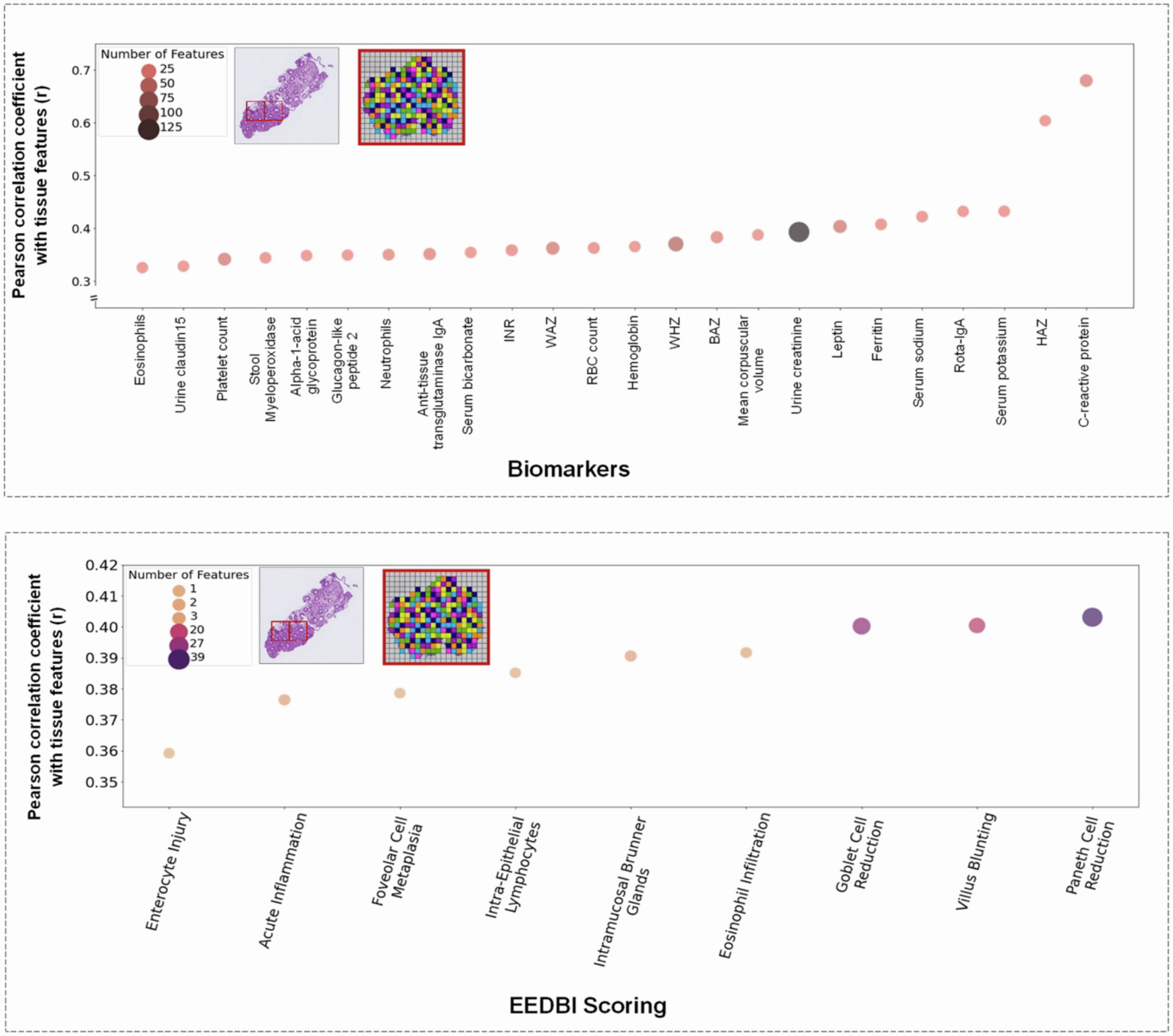

Figure S1: Numerical tissue feature representation correlations with clinical biomarkers and EEDBI Scores. **Related to Figure 2**

Table S1: Results of Functional Enrichment Analysis using ToppGene, related to Figure 2

Category	ID	Name	Source	p-value	q-value Bonferroni	q-value FDR B&H	q-value FDR B&Y	Hit Count in Query List	Hit Count in Genome	Hit in Query List
Pathway	132956	Metabolic pathways	BioSystems: KEGG	8.62E-05	4.75E-02	0.01	8.18E-02	16	1272	CMBL,ADH6,HGD,UPB1,ACAT1,AMACR,AKR1B10,SHMT1,OAT,CYP4F3,ADI1,FTCD,ALDOB,ME1,MOCS1,ADH4
Pathway	1270158	Metabolism of amino acids and derivatives	BioSystems: REACTOME	2.97E-05	1.64E-02	0.01	5.12E-02	9	367	HGD,ACAT1,SLC25A15,IYD,SHMT1,OAT,DDO,ADI1,FTCD
Pathway	1270190	Phase 1 - Functionalization of compounds	BioSystems: REACTOME	1.25E-04	6.90E-02	0.01	9.51E-02	5	111	CMBL,ADH6,EPHX1,CYP4F3,ADH4
Pathway	1270144	Metabolism of vitamins and cofactors	BioSystems: REACTOME	1.07E-03	5.90E-01	0.03	2.41E-01	5	177	LRAT,AKR1B10,SHMT1,MOCS1,CYB5A
Pathway	M699	Fatty acid metabolism	MSigDB C2 BIOCARTA (v7.3)	3.36E-05	1.85E-02	0.01	5.12E-02	4	42	ADH6,ACAT1,ECI2,ADH4
Pathway	82935	Fatty acid degradation	BioSystems: KEGG	4.04E-05	2.23E-02	0.01	5.12E-02	4	44	ADH6,ACAT1,ECI2,ADH4
Pathway	M16794	Metabolism of xenobiotics by cytochrome P450	MSigDB C2 BIOCARTA (v7.3)	2.51E-04	1.38E-01	0.02	1.07E-01	4	70	ADH6,EPHX1,DHDH,ADH4
Pathway	193146	Bile secretion	BioSystems: KEGG	2.65E-04	1.46E-01	0.02	1.07E-01	4	71	EPHX1,SLC10A2,CFTR,CA2
Pathway	83031	Metabolism of xenobiotics by cytochrome P450	BioSystems: KEGG	3.11E-04	1.71E-01	0.02	1.07E-01	4	74	ADH6,EPHX1,DHDH,ADH4
Pathway	814926	Carbon metabolism	BioSystems: KEGG	1.58E-03	8.71E-01	0.04	3.00E-01	4	114	ACAT1,SHMT1,ALDOB,ME1
Pathway	1270031	Peroxisomal lipid metabolism	BioSystems: REACTOME	2.73E-04	1.51E-01	0.02	1.07E-01	3	29	ACBD4,AMACR,ECI2
Pathway	MAP00350_Tyrosine_metabolism	MAP00350 Tyrosine metabolism	GenMAPP	3.03E-04	1.67E-01	0.02	1.07E-01	3	30	ADH6,HGD,ADH4
Pathway	82959	Tyrosine metabolism	BioSystems: KEGG	4.81E-04	2.65E-01	0.02	1.52E-01	3	35	ADH6,HGD,ADH4
Pathway	M16743	Tyrosine metabolism	MSigDB C2 BIOCARTA (v7.3)	8.25E-04	4.54E-01	0.03	2.12E-01	3	42	ADH6,HGD,ADH4
Pathway	MAP00071_Fatty_acid_metabolism	MAP00071 Fatty acid metabolism	GenMAPP	1.08E-03	5.93E-01	0.03	2.41E-01	3	46	ADH6,ACAT1,ADH4
Pathway	MAP00010_Glycolysis_Gluconeogenesis	MAP00010 Glycolysis Gluconeogenesis	GenMAPP	1.54E-03	8.48E-01	0.04	3.00E-01	3	52	ADH6,ALDOB,ADH4
Pathway	M39326	Benzene metabolism	MSigDB C2 BIOCARTA (v7.3)	2.83E-04	1.56E-01	0.02	1.07E-01	2	6	EPHX1,DHDH
Pathway	545354	noradrenaline and adrenaline degradation	BioSystems: BIOCYC	6.73E-04	3.71E-01	0.03	1.97E-01	2	9	ADH6,ADH4
Pathway	SMP00053	One Carbon Pool By Folate	SMPDB	8.39E-04	4.62E-01	0.03	2.12E-01	2	10	SHMT1,FTCD
Pathway	1270206	Ethanol oxidation	BioSystems: REACTOME	1.22E-03	6.74E-01	0.04	2.58E-01	2	12	ADH6,ADH4
Pathway	PW:0000189	folate mediated one-carbon metabolic	Pathway Ontology	1.93E-03	1.00E+00	0.05	3.33E-01	2	15	SHMT1,FTCD

Pathway	M39717	Fatty Acid Omega Oxidation	MSigDB C2 BIOCARTA (v7.3)	1.93E-03	1.00E+00	0.05	3.33E-01	2	15	ADH6,ADH4
Human Phenotype	HP:0004354	Abnormal circulating carboxylic acid concentration		2.76E-06	3.15E-03	0.00	2.40E-02	9	290	EPHX1,HGD,SLC25A15,CFTR,OAT,FTCD,ALDOB,MOCS1,CYB5A
Human Phenotype	HP:0004337	Abnormality of amino acid metabolism		2.48E-05	2.84E-02	0.01	5.41E-02	8	285	HGD,SLC25A15,CFTR,OAT,FTCD,ALDOB,MOCS1,CYB5A
Human Phenotype	HP:0002630	Fat malabsorption		1.47E-04	1.68E-01	0.03	2.56E-01	4	60	EPHX1,SLC10A2,AMACR,CFTR
Human Phenotype	HP:0012025	Abnormal circulating ornithine concentration		2.10E-05	2.39E-02	0.01	5.41E-02	2	2	SLC25A15,OAT
Human Phenotype	HP:0012026	Hyperornithinemia		2.10E-05	2.39E-02	0.01	5.41E-02	2	2	SLC25A15,OAT
GO: Molecular Function	GO:0016491	oxidoreductase activity		1.14E-05	4.73E-03	0.00	3.12E-02	13	783	ADH6,HGD,SELENOW,FMO5,DHDH,AKR1B10,IYD,DDO,CYP4F3,ADI1,ME1,CYB5A,ADH4
GO: Molecular Function	GO:0046914	transition metal ion binding		1.57E-03	6.49E-01	0.04	2.59E-01	12	1130	ADH6,MMP24,UPB1,SMPDL3B,MMP28,SHMT1,CYP4F3,ADAMTS13,ADI1,ME1,CA2,ADH4
GO: Molecular Function	GO:0008270	zinc ion binding		6.32E-03	1.00E+00	0.05	3.26E-01	9	849	ADH6,MMP24,UPB1,SMPDL3B,MMP28,SHMT1,ADAMTS13,CA2,ADH4
GO: Molecular Function	GO:0016829	lyase activity		1.92E-04	7.93E-02	0.02	1.05E-01	6	211	SHMT1,FTCD,ALDOB,ME1,MOCS1,CA2
GO: Molecular Function	GO:0016616	oxidoreductase activity, acting on the CH-OH group of donors, NAD or NADP as acceptor		1.39E-04	5.77E-02	0.02	1.05E-01	5	125	ADH6,DHDH,AKR1B10,ME1,ADH4
GO: Molecular Function	GO:0016614	oxidoreductase activity, acting on CH-OH group of donors		1.93E-04	7.98E-02	0.02	1.05E-01	5	134	ADH6,DHDH,AKR1B10,ME1,ADH4
GO: Molecular Function	GO:0019842	vitamin binding		3.56E-04	1.48E-01	0.02	1.39E-01	5	153	LRAT,SHMT1,OAT,FTCD,ADH4
GO: Molecular Function	GO:0016830	carbon-carbon lyase activity		7.70E-05	3.19E-02	0.02	1.05E-01	4	57	SHMT1,ALDOB,ME1,MOCS1
GO: Molecular Function	GO:0046943	carboxylic acid transmembrane transporter activity		3.77E-03	1.00E+00	0.04	2.59E-01	4	160	SLC26A3,SLC10A2,SLC25A15,SLC13A2
GO: Molecular Function	GO:0005342	organic acid transmembrane transporter activity		3.85E-03	1.00E+00	0.04	2.59E-01	4	161	SLC26A3,SLC10A2,SLC25A15,SLC13A2
GO: Molecular Function	GO:0016853	isomerase activity		4.77E-03	1.00E+00	0.04	2.97E-01	4	171	AMACR,CFTR,PBLD,ECI2
GO: Molecular Function	GO:0008514	organic anion transmembrane transporter activity		6.05E-03	1.00E+00	0.05	3.26E-01	4	183	SLC26A3,SLC25A15,CFTR,SLC13A2
GO: Molecular Function	GO:0016903	oxidoreductase activity, acting on the aldehyde or oxo group of donors		8.58E-04	3.55E-01	0.03	1.96E-01	3	47	FMO5,AKR1B10,ADH4
GO: Molecular Function	GO:0015370	solute:sodium symporter activity		3.43E-03	1.00E+00	0.04	2.59E-01	3	76	SLC10A2,SLC13A2,SLC5A4
GO: Molecular Function	GO:0004024	alcohol dehydrogenase activity, zinc-dependent		3.22E-04	1.33E-01	0.02	1.39E-01	2	7	ADH6,ADH4
GO: Molecular Function	GO:0016742	hydroxymethyl-, formyl- and related transferase activity		4.28E-04	1.77E-01	0.02	1.46E-01	2	8	SHMT1,FTCD
GO: Molecular Function	GO:0004022	alcohol dehydrogenase (NAD+) activity		5.49E-04	2.27E-01	0.03	1.67E-01	2	9	ADH6,ADH4

GO: Molecular Function	GO:0018455	alcohol dehydrogenase [NAD(P)+] activity	6.84E-04	2.83E-01	0.03	1.87E-01	2	10	ADH6,ADH4
GO: Molecular Function	GO:0016832	aldehyde-lyase activity	8.34E-04	3.45E-01	0.03	1.96E-01	2	11	SHMT1,ALDOB
GO: Molecular Function	GO:0004032	alditol:NADP+ 1-oxidoreductase activity	9.99E-04	4.13E-01	0.03	2.10E-01	2	12	AKR1B10,ADH4
GO: Molecular Function	GO:0019841	retinol binding	1.80E-03	7.44E-01	0.04	2.59E-01	2	16	LRAT,ADH4
GO: Molecular Function	GO:0015106	bicarbonate transmembrane transporter activity	3.11E-03	1.00E+00	0.04	2.59E-01	2	21	SLC26A3,CFTR
GO: Molecular Function	GO:0008106	alcohol dehydrogenase (NADP+) activity	3.11E-03	1.00E+00	0.04	2.59E-01	2	21	AKR1B10,ADH4
GO: Molecular Function	GO:0004745	NAD-retinol dehydrogenase activity	3.11E-03	1.00E+00	0.04	2.59E-01	2	21	ADH6,ADH4
GO: Molecular Function	GO:0000062	fatty-acyl-CoA binding	4.74E-03	1.00E+00	0.04	2.97E-01	2	26	ACBD4,ECI2
GO: Molecular Function	GO:0004033	aldo-keto reductase (NADP) activity	5.11E-03	1.00E+00	0.05	3.04E-01	2	27	AKR1B10,ADH4
GO: Molecular Function	GO:0016702	oxidoreductase activity, acting on single donors with incorporation of molecular oxygen, incorporation of two atoms of oxygen	5.11E-03	1.00E+00	0.05	3.04E-01	2	27	HGD,ADI1
GO: Molecular Function	GO:1901567	fatty acid derivative binding	5.49E-03	1.00E+00	0.05	3.13E-01	2	28	ACBD4,ECI2
GO: Molecular Function	GO:0016701	oxidoreductase activity, acting on single donors with incorporation of molecular oxygen	5.49E-03	1.00E+00	0.05	3.13E-01	2	28	HGD,ADI1
GO: Molecular Function	GO:0005343	organic acid:sodium symporter activity	5.88E-03	1.00E+00	0.05	3.26E-01	2	29	SLC10A2,SLC13A2
GO: Molecular Function	GO:0005310	dicarboxylic acid transmembrane transporter activity	6.29E-03	1.00E+00	0.05	3.26E-01	2	30	SLC26A3,SLC13A2
GO: Molecular Function	GO:0120227	acyl-CoA binding	6.29E-03	1.00E+00	0.05	3.26E-01	2	30	ACBD4,ECI2
GO: Molecular Function	GO:0004411	homogentisate 1,2-dioxygenase activity	3.98E-03	1.00E+00	0.04	2.59E-01	1	1	HGD
GO: Molecular Function	GO:0047173	phosphatidylcholine-retinol O-acyltransferase activity	3.98E-03	1.00E+00	0.04	2.59E-01	1	1	LRAT
GO: Molecular Function	GO:0015361	low-affinity sodium:dicarboxylate symporter activity	3.98E-03	1.00E+00	0.04	2.59E-01	1	1	SLC13A2
GO: Molecular Function	GO:0004587	ornithine-oxo-acid transaminase activity	3.98E-03	1.00E+00	0.04	2.59E-01	1	1	OAT
GO: Molecular Function	GO:0106138	Sec61 translocon complex binding	3.98E-03	1.00E+00	0.04	2.59E-01	1	1	CFTR
GO: Molecular Function	GO:0061798	GTP 3',8'-cyclase activity	3.98E-03	1.00E+00	0.04	2.59E-01	1	1	MOCS1
GO: Molecular Function	GO:0061799	cyclic pyranopterin monophosphate synthase activity	3.98E-03	1.00E+00	0.04	2.59E-01	1	1	MOCS1
GO: Molecular Function	GO:0030412	formimidoyltetrahydrofolate cyclodeaminase activity	3.98E-03	1.00E+00	0.04	2.59E-01	1	1	FTCD

GO: Molecular Function	GO:0050155	ornithine(lysine) transaminase activity	3.98E-03	1.00E+00	0.04	2.59E-01	1	1	OAT
GO: Molecular Function	GO:0030409	glutamate formimidoyltransferase activity	3.98E-03	1.00E+00	0.04	2.59E-01	1	1	FTCD
GO: Molecular Function	GO:0033961	cis-stilbene-oxide hydrolase activity	3.98E-03	1.00E+00	0.04	2.59E-01	1	1	EPHX1
GO: Molecular Function	GO:0005260	intracellularly ATP-gated chloride channel activity	3.98E-03	1.00E+00	0.04	2.59E-01	1	1	CFTR
GO: Molecular Function	GO:0008111	alpha-methylacyl-CoA racemase activity	3.98E-03	1.00E+00	0.04	2.59E-01	1	1	AMACR
GO: Molecular Function	GO:0047837	D-xylose 1-dehydrogenase (NADP+) activity	3.98E-03	1.00E+00	0.04	2.59E-01	1	1	DHDH
GO: Molecular Function	GO:0010309	acireductone dioxygenase [iron(II)-requiring] activity	3.98E-03	1.00E+00	0.04	2.59E-01	1	1	ADI1
GO: Molecular Function	GO:0099142	intracellularly ATP-gated ion channel activity	3.98E-03	1.00E+00	0.04	2.59E-01	1	1	CFTR
GO: Molecular Function	GO:0008445	D-aspartate oxidase activity	3.98E-03	1.00E+00	0.04	2.59E-01	1	1	DDO
GO: Molecular Function	GO:0061609	fructose-1-phosphate aldolase activity	3.98E-03	1.00E+00	0.04	2.59E-01	1	1	ALDOB
GO: Molecular Function	GO:0102279	lecithin:11-cis retinol acyltransferase activity	3.98E-03	1.00E+00	0.04	2.59E-01	1	1	LRAT
GO: Molecular Function	GO:0035538	carbohydrate response element binding	3.98E-03	1.00E+00	0.04	2.59E-01	1	1	MLXIPL
GO: Molecular Function	GO:0015922	aspartate oxidase activity	3.98E-03	1.00E+00	0.04	2.59E-01	1	1	DDO
GO: Biological Process	GO:0019752	carboxylic acid metabolic process	1.20E-08	1.93E-05	0.00	7.70E-05	19	1064	ADH6,EPHX1,HGD,MLXIPL,UPB1,ACAT1,AMACR,IYD,SHMT1,OAT,DDO,CYP4F3,ECI2,ADI1,FTCD,ALDOB,ME1,CYB5A,ADH4
GO: Biological Process	GO:0043436	oxoacid metabolic process	1.98E-08	3.21E-05	0.00	8.51E-05	19	1098	ADH6,EPHX1,HGD,MLXIPL,UPB1,ACAT1,AMACR,IYD,SHMT1,OAT,DDO,CYP4F3,ECI2,ADI1,FTCD,ALDOB,ME1,CYB5A,ADH4
GO: Biological Process	GO:0006082	organic acid metabolic process	3.14E-08	5.07E-05	0.00	1.01E-04	19	1130	ADH6,EPHX1,HGD,MLXIPL,UPB1,ACAT1,AMACR,IYD,SHMT1,OAT,DDO,CYP4F3,ECI2,ADI1,FTCD,ALDOB,ME1,CYB5A,ADH4
GO: Biological Process	GO:0006629	lipid metabolic process	1.04E-05	1.68E-02	0.00	1.45E-02	18	1503	ADH6,EPHX1,LRAT,MLXIPL,CPNE6,FMO5,SMPDL3B,ACAT1,AMACR,CFTR,AKR1B10,ISX,CYP4F3,ECI2,APOH,ME1,CYB5A,ADH4
GO: Biological Process	GO:0044255	cellular lipid metabolic process	1.55E-05	2.50E-02	0.00	1.81E-02	15	1106	ADH6,EPHX1,LRAT,MLXIPL,CPNE6,SMPDL3B,ACAT1,AMACR,AKR1B10,ISX,CYP4F3,ECI2,APOH,CYB5A,ADH4
GO: Biological Process	GO:0044282	small molecule catabolic process	1.49E-09	2.40E-06	0.00	1.92E-05	14	452	HGD,UPB1,DHDH,ACAT1,AMACR,AKR1B10,SHMT1,OAT,DDO,CYP4F3,ECI2,FTCD,ALDOB,ADH4
GO: Biological Process	GO:0032787	monocarboxylic acid metabolic process	1.13E-05	1.82E-02	0.00	1.45E-02	12	692	ADH6,EPHX1,MLXIPL,ACAT1,AMACR,CYP4F3,ECI2,FTCD,ALDOB,ME1,CYB5A,ADH4
GO: Biological Process	GO:1901615	organic hydroxy compound metabolic process	1.08E-04	1.74E-01	0.01	7.30E-02	10	613	ADH6,EPHX1,LRAT,FMO5,AMACR,CFTR,AKR1B10,IYD,CYP4F3,ADH4
GO: Biological Process	GO:0044283	small molecule biosynthetic process	5.03E-04	8.12E-01	0.03	2.04E-01	10	743	EPHX1,MLXIPL,UPB1,ACAT1,AMACR,CFTR,SHMT1,OAT,ADI1,ALDOB
GO: Biological Process	GO:0046395	carboxylic acid catabolic process	4.71E-07	7.60E-04	0.00	1.21E-03	9	251	HGD,ACAT1,AMACR,SHMT1,OAT,DDO,CYP4F3,ECI2,FTCD

GO: Biological Process	GO:0016054	organic acid catabolic process	7.64E-07	1.24E-03	0.00	1.64E-03	9	266	HGD,ACAT1,AMACR,SHMT1,OAT,DDO,CYP4F3,ECI2,FTCD
GO: Biological Process	GO:0006520	cellular amino acid metabolic process	6.44E-06	1.04E-02	0.00	1.04E-02	9	345	HGD,UPB1,ACAT1,IYD,SHMT1,OAT,DDO,ADI1,FTCD
GO: Biological Process	GO:0010817	regulation of hormone levels	5.58E-04	9.01E-01	0.03	2.11E-01	9	618	ADH6,LRAT,NELL2,CFTR,AKR1B10,IYD,DDO,BAIAP3,ADH4
GO: Biological Process	GO:1901605	alpha-amino acid metabolic process	1.21E-06	1.96E-03	0.00	2.23E-03	8	207	HGD,ACAT1,IYD,SHMT1,OAT,DDO,ADI1,FTCD
GO: Biological Process	GO:0006631	fatty acid metabolic process	1.92E-04	3.11E-01	0.01	1.13E-01	8	419	EPHX1,MLXIPL,ACAT1,AMACR,CYP4F3,ECI2,CYB5A,ADH4
GO: Biological Process	GO:0006091	generation of precursor metabolites and energy	1.47E-03	1.00E+00	0.04	3.57E-01	8	571	ADH6,MLXIPL,DHDH,ACAT1,ALDOB,ME1,CYB5A,ADH4
GO: Biological Process	GO:0016042	lipid catabolic process	4.07E-04	6.58E-01	0.02	1.81E-01	7	355	SMPDL3B,ACAT1,AMACR,AKR1B10,CYP4F3,ECI2,APOH
GO: Biological Process	GO:0006066	alcohol metabolic process	9.26E-04	1.00E+00	0.03	2.71E-01	7	408	ADH6,EPHX1,LRAT,FMO5,CFTR,AKR1B10,ADH4
GO: Biological Process	GO:0006720	isoprenoid metabolic process	1.99E-05	3.22E-02	0.00	2.14E-02	6	146	ADH6,LRAT,AMACR,AKR1B10,ISX,ADH4
GO: Biological Process	GO:0044242	cellular lipid catabolic process	3.52E-04	5.69E-01	0.02	1.68E-01	6	246	SMPDL3B,ACAT1,AMACR,AKR1B10,ECI2,APOH
GO: Biological Process	GO:0042445	hormone metabolic process	3.92E-04	6.34E-01	0.02	1.80E-01	6	251	ADH6,LRAT,AKR1B10,IYD,DDO,ADH4
GO: Biological Process	GO:0009636	response to toxic substance	4.44E-04	7.18E-01	0.02	1.91E-01	6	257	EPHX1,SELENOW,AKR1B10,IYD,ADAMTS13,ADH4
GO: Biological Process	GO:1901606	alpha-amino acid catabolic process	2.40E-05	3.88E-02	0.00	2.38E-02	5	90	HGD,ACAT1,SHMT1,OAT,DDO
GO: Biological Process	GO:0009063	cellular amino acid catabolic process	5.28E-05	8.53E-02	0.01	4.53E-02	5	106	HGD,SHMT1,OAT,DDO,FTCD
GO: Biological Process	GO:0043648	dicarboxylic acid metabolic process	5.77E-05	9.33E-02	0.01	4.57E-02	5	108	SHMT1,OAT,DDO,FTCD,ME1
GO: Biological Process	GO:0001523	retinoid metabolic process	6.03E-05	9.75E-02	0.01	4.57E-02	5	109	ADH6,LRAT,AKR1B10,ISX,ADH4
GO: Biological Process	GO:0016101	diterpenoid metabolic process	7.79E-05	1.26E-01	0.01	5.57E-02	5	115	ADH6,LRAT,AKR1B10,ISX,ADH4
GO: Biological Process	GO:0006721	terpenoid metabolic process	1.16E-04	1.87E-01	0.01	7.43E-02	5	125	ADH6,LRAT,AKR1B10,ISX,ADH4
GO: Biological Process	GO:0006766	vitamin metabolic process	1.72E-04	2.77E-01	0.01	1.05E-01	5	136	LRAT,SHMT1,ISX,CYP4F3,CYB5A
GO: Biological Process	GO:0042572	retinol metabolic process	3.34E-05	5.39E-02	0.00	3.07E-02	4	48	ADH6,LRAT,AKR1B10,ADH4
GO: Biological Process	GO:0008652	cellular amino acid biosynthetic process	2.59E-04	4.19E-01	0.02	1.39E-01	4	81	UPB1,SHMT1,OAT,ADI1
GO: Biological Process	GO:0034308	primary alcohol metabolic process	5.79E-04	9.35E-01	0.03	2.13E-01	4	100	ADH6,LRAT,AKR1B10,ADH4
GO: Biological Process	GO:0006885	regulation of pH	9.77E-04	1.00E+00	0.03	2.74E-01	4	115	SLC26A3,CFTR,CHP2,CA2

GO: Biological Process	GO:0019395	fatty acid oxidation	9.77E-04	1.00E+00	0.03	2.74E-01	4	115	ACAT1,AMACR,ECI2,ADH4
GO: Biological Process	GO:1990748	cellular detoxification	1.01E-03	1.00E+00	0.03	2.76E-01	4	116	SELENOW,AKR1B10,IYD,ADH4
GO: Biological Process	GO:0034440	lipid oxidation	1.15E-03	1.00E+00	0.04	3.07E-01	4	120	ACAT1,AMACR,ECI2,ADH4
GO: Biological Process	GO:0097237	cellular response to toxic substance	1.25E-03	1.00E+00	0.04	3.19E-01	4	123	SELENOW,AKR1B10,IYD,ADH4
GO: Biological Process	GO:1901661	quinone metabolic process	3.19E-04	5.15E-01	0.02	1.58E-01	3	35	AKR1B10,CYP4F3,ADH4
GO: Biological Process	GO:0006730	one-carbon metabolic process	5.48E-04	8.86E-01	0.03	2.11E-01	3	42	SHMT1,FTCD,CA2
GO: Biological Process	GO:0015701	bicarbonate transport	6.29E-04	1.00E+00	0.03	2.14E-01	3	44	SLC26A3,CFTR,CA2
GO: Biological Process	GO:0006775	fat-soluble vitamin metabolic process	8.63E-04	1.00E+00	0.03	2.71E-01	3	49	LRAT,ISX,CYP4F3
GO: Biological Process	GO:0051454	intracellular pH elevation	2.13E-04	3.44E-01	0.02	1.19E-01	2	6	SLC26A3,CFTR
GO: Biological Process	GO:0045852	pH elevation	2.97E-04	4.80E-01	0.02	1.53E-01	2	7	SLC26A3,CFTR
GO: Biological Process	GO:0110095	cellular detoxification of aldehyde	5.07E-04	8.19E-01	0.03	2.04E-01	2	9	AKR1B10,ADH4
GO: Biological Process	GO:0006776	vitamin A metabolic process	6.32E-04	1.00E+00	0.03	2.14E-01	2	10	LRAT,ISX
GO: Biological Process	GO:0046073	dTMP metabolic process	6.32E-04	1.00E+00	0.03	2.14E-01	2	10	UPB1,SHMT1
GO: Biological Process	GO:2001225	regulation of chloride transport	7.71E-04	1.00E+00	0.03	2.48E-01	2	11	CFTR,CA2
GO: Biological Process	GO:0008300	isoprenoid catabolic process	7.71E-04	1.00E+00	0.03	2.48E-01	2	11	AMACR,AKR1B10
GO: Biological Process	GO:0035999	tetrahydrofolate interconversion	9.22E-04	1.00E+00	0.03	2.71E-01	2	12	SHMT1,FTCD
GO: Biological Process	GO:0006069	ethanol oxidation	9.22E-04	1.00E+00	0.03	2.71E-01	2	12	ADH6,ADH4
GO: Biological Process	GO:0110096	cellular response to aldehyde	1.27E-03	1.00E+00	0.04	3.19E-01	2	14	AKR1B10,ADH4
GO: Biological Process	GO:0006570	tyrosine metabolic process	1.27E-03	1.00E+00	0.04	3.19E-01	2	14	HGD,IYD
GO: Biological Process	GO:0033540	fatty acid beta-oxidation using acyl-CoA oxidase	1.46E-03	1.00E+00	0.04	3.57E-01	2	15	AMACR,ECI2
GO: Biological Process	GO:1901569	fatty acid derivative catabolic process	1.66E-03	1.00E+00	0.05	3.82E-01	2	16	ACAT1,CYP4F3
GO: Biological Process	GO:0034392	negative regulation of smooth muscle cell apoptotic process	1.66E-03	1.00E+00	0.05	3.82E-01	2	16	CFTR,APOH
GO: Biological Process	GO:0009176	pyrimidine deoxyribonucleoside monophosphate metabolic process	1.66E-03	1.00E+00	0.05	3.82E-01	2	16	UPB1,SHMT1

Gene Family	752	Solute carriers	genenames.org	2.55E-03	1.07E-01	0.03	1.16E-01	5	395	SLC26A3,SLC10A2,SLC25A15,SLC13A2,SLC5A4
Gene Family	397	Alcohol dehydrogenases	genenames.org	1.59E-04	6.66E-03	0.01	2.88E-02	2	8	ADH6,ADH4
Gene Family	891	Matrix metallopeptidases	genenames.org	1.53E-03	6.41E-02	0.03	1.16E-01	2	24	MMP24,MMP28
Gene Family	309	Chloride channels, ATP-gated CFTR|ATP binding cassette subfamily C	genenames.org	2.42E-03	1.02E-01	0.03	1.16E-01	1	1	CFTR

**Table S2: Reactions with altered flux in Pakistan cohort, related to Figure 3**				
**Reaction ID**	**Chemical Formula of Reaction**	**System**	**Description**	
**HMR_2296**	h[c] + nadph[c] + o2[c] + stcoa[c] -> 2.0 h2o[c] + M00116[c] + nadp[c]	**Fatty acid synthesis**	Stearoyl Coenzyme A 9-Desaturase	
**CDIPTr**	cdpdag_hs[c] + inost[c] <=> cmp[c] + h[c] + pail_hs[c]	Glycerophospholipid metabolism	Phosphatidylinositol Synthase	
**r1431**	dadp[m] + h2o[m] + trdox[m] -> adp[m] + trdrd[m]	Oxidoreductase	2-Deoxyuridine 5-Diphosphate:Oxidized-Thioredoxin 2-Oxidoreductase	ribonucleotide reductase
**sink_arg_L[c]**	arg_L[c] <=>	Exchange/demand reaction		
**sink_lnlncacoa[c]**	lnlncacoa[c] <=>	Linoleic acid metabolism	Exchange/demand reaction	Exchange of alpha-Linolenoyl-CoA
**HMR_0279**	atp[c] + coa[c] + M02613[c] <=> amp[c] + M02612[c] + ppi[c]	**Fatty acid synthesis**	Long-Chain-Fatty-Acid Coenzyme A Ligase	acyl-CoA synthetase long-chain family member
**PAIL_hs_t1e**	pail_hs[e] -> pail_hs[c]	Glycerophospholipid metabolism	Transport of 1-Phosphatidyl-1D-Myo-Inositol, Vescicular	
**EX_pail_hs[e]**	pail_hs[e] <=>	Glycerophospholipid metabolism	Exchange of 1-Phosphatidyl-1D-Myo-Inositol	
**HMR_0241**	atp[c] + coa[c] + M00003[c] <=> amp[c] + M00004[c] + ppi[c]	**Fatty acid synthesis**	Long-Chain-Fatty-Acid Coenzyme A Ligase	acyl-CoA synthetase long-chain family member
**HMR_0385**	atp[c] + coa[c] + M00341[c] <=> amp[c] + M00343[c] + ppi[c]	**Fatty acid synthesis**	Long-Chain-Fatty-Acid Coenzyme A Ligase	
HMR_0293	atp[c] + CE2510[c] + coa[c] <=> amp[c] + CE5151[c] + ppi[c]	Fatty acid synthesis	Long-Chain-Fatty-Acid Coenzyme A Ligase	
RE3446C	atp[c] + CE4987[c] + coa[c] <=> amp[c] + CE5969[c] + ppi[c]	Eicosanoid metabolism	Long-Chain-Fatty-Acid- Coenzyme A Ligase	
HMR_0192	atp[c] + coa[c] + M03051[c] <=> amp[c] + M03050[c] + ppi[c]	Fatty acid synthesis	Long-Chain-Fatty-Acid Coenzyme A Ligase	
HMR_1267	CE5969[c] -> CE5969[m]	Transport	Mitochondrial transport	
HMR_0245	atp[c] + coa[c] + M01238[c] <=> amp[c] + M01237[c] + ppi[c]	Fatty acid synthesis	Long-Chain-Fatty-Acid Coenzyme A Ligase	
HMR_1284	CE4988[c] <=> CE4988[m]	Transport	Mitochondrial transport	
RE3432C	CE5944[c] + h[c] + nadph[c] <=> CE4987[c] + nadp[c]	Eicosanoid metabolism	Carbonyl Reductase (NADPH)	
NADH2_u10mi	5.0 h[m] + nadh[m] + q10[m] -> 4.0 h[i] + nad[m] + q10h2[m]	Oxidative phosphorylation		
EX_lnlccrn[e]	lnlccrn[e] <=>	Exchange/demand reaction	Exchange of Linoleyl Carnitine	
HMR_0309	atp[c] + coa[c] + M02053[c] <=> amp[c] + M02052[c] + ppi[c]	Fatty acid synthesis	Long-Chain-Fatty-Acid Coenzyme A Ligase	

Table S3: Reactions with altered flux in Zambia cohort, related to Figure 3

**HMR_0319**	atp[c] + coa[c] + doco13ac[c] <=> amp[c] + CE5155[c] + ppi[c]	Fatty acid synthesis	Long-Chain-Fatty-Acid Coenzyme A Ligase
**RE3239C**	CE5155[c] + h2o[c] <=> coa[c] + doco13ac[c] + h[c]	Fatty acid synthesis	
**biomass_reaction**	long	Exchange/ demand	Generic Human Biomass Reaction
**HMR_0293**	atp[c] + CE2510[c] + coa[c] <=> amp[c] + CE5151[c] + ppi[c]	Fatty acid synthesis	Long-Chain-Fatty-Acid Coenzyme A Ligase
**PAIL_HStn**	pail_hs[c] <=> pail_hs[n]	Glycerophospholipid metabolism	Phosphatidylinositol Nuclear Transport (Diffusion)
**HMR_9187**	M01966[c] <=> M01966[e]	Starch and sucrose metabolism	Extracellular Transport of Alpha-D-Glucose 1,6-bisphosphate
**HMR_8585**	13dpg[c] + g1p[c] -> 3pg[c] + M01966[c]	Starch and sucrose metabolism	3-Phospho-D-Glyceroyl-Phosphate:Alpha-D-Glucose-1-Phosphate 6-Phosphotransferase
**EX_M01966[e]**		Starch and sucrose metabolism	Alpha-D-Glucose 1,6-bisphosphate
**EX_pail_hs[e]**	pail_hs[e] <=>	Glycerophospholipid metabolism	Exchange of 1-Phosphatidyl-1D-Myo-Inositol
**PAIL_hs_t1e**	pail_hs[e] -> pail_hs[c]	Glycerophospholipid metabolism	Vesicular Transport of 1-Phosphatidyl-1D-Myo-Inositol
**r0430**	crn[r] + h[r] + malcoa[r] -> coa[r] + HC10859[r]	Fatty acid oxidation	Palmitoyl Coenzyme A:L-Carnitine O-Palmitoyltransferase
**r1004**	crn[c] + HC10859[r] -> crn[r] + HC10859[c]	Transport, endoplasmic reticular	Facilitated Diffusion
**GLGNS1**	ggn[c] + 3.0 udpg[c] -> glygn1[c] + 3.0 h[c] + 3.0 udp[c]	Starch and sucrose metabolism	Glycogen Synthase (Ggn -> Glygn1)
**GLBRAN**	glygn1[c] -> glygn2[c]	Starch and sucrose metabolism	1, 4-Alpha-Glucan Branching Enzyme (Glygn1 -> Glygn2)
**GALU**	g1p[c] + h[c] + utp[c] <=> ppi[c] + udpg[c]	Galactose metabolism	UTP-Glucose-1-Phosphate Uridylyltransferase
**DM_mi145p[c]**	mi145p[c] ->	Exchange/ demand	Demand for 1D-Myo-Inositol 1,4,5-Trisphosphate
**DTMPKm**	atp[m] + dtmp[m] -> adp[m] + dtdp[m]	Pyrimidine synthesis	DTMP Kinase in Mitochondria
**GLPASE1**	glygn2[c] + 3.0 pi[c] -> dxtrn[c] + 3.0 g1p[c]	Starch and sucrose metabolism	Glycogen Phosphorylase (Glygn2 -> Dxtrn)
**PI45PLC**	h2o[c] + pail45p_hs[c] -> dag_hs[c] + h[c] + mi145p[c]	Inositol phosphate metabolism	Phosphatidylinositol 4, 5-Bisphosphate Phospholipase C
**RE1447N**	h2o[n] + pail45p_hs[n] <=> pail5p_hs[n] + pi[n]	Phosphatidylinositol phosphate metabolism	Phosphatidylinositol-3,4-Bisphosphate 4-Phosphatase

Table S4. Lipid molecules differentially expressed in plasma of EED children in positive ion data
Related to Figure 4

Lipid name	Fold change (EED/Normal)	*p* value	q value (FDR adjusted)
CE 18:3	-2.7	1.97E-03	0.006
TG 54:8	-2.0	1.06E-04	0.001
PG 40:6	-2.2	2.93E-03	0.009
LPS 22:0	-3.7	8.00E-04	0.003
CE(8D5)	-3.0	5.74E-04	0.003
CE(9M6)	-3.0	5.48E-04	0.003
CE(11D4)	-3.1	9.44E-04	0.004
CE(12D5)	-2.8	4.08E-04	0.002
CE(15D3)	-2.9	3.92E-04	0.002
8F6 (Furocarpic acid)	-2.7	6.79E-04	0.003
AC(13:0(3-OH))	-3.0	1.84E-03	0.006
Cer(d18:0/23:0)	3.4	6.71E-08	<0.001
DG(18:0/18:1)	2.9	1.33E-05	<0.001
LPE 16:1	3.0	3.80E-04	0.002
LPE O-16:1	2.6	2.60E-10	<0.001
PC 29:0	3.2	1.51E-02	0.034
PC 31:0	2.8	2.17E-03	0.007
PE 34:1	2.5	1.66E-03	0.006
PE 34:2	2.6	1.12E-03	0.004
PE 36:4	2.4	8.99E-04	0.004
PE O-36:4	5.6	1.87E-07	<0.001
PI 40:6	2.8	5.28E-04	0.002
TG 44:3	4.2	1.52E-02	0.034
TG 46:1	2.9	6.08E-03	0.016
TG 46:2	2.4	8.31E-03	0.021
TG 46:3	2.3	2.21E-03	0.007
TG 48:1	2.5	4.15E-05	<0.001
TG 49:1	2.3	3.21E-05	<0.001
TG 50:1	2.0	3.40E-12	<0.001
TG 51:1	2.0	8.57E-06	<0.001
TG O-50:1	3.8	6.18E-05	<0.001
TG O-50:2	3.1	1.79E-07	<0.001
TG O-52:2	2.6	2.33E-04	0.001

*33 (out of 201) positive ion lipid features (fold change > 2 & FDR adjusted q-value < 0.05) between AKU and CCHMC control, among them 11 significantly down-regulated in AKU (represented as negative value), 22 significantly up-regulated in AKU. Fold change colored in blue indicates a decrease of EED children compared to their normal counterpart, red indicate an increase.

Lipid class abbreviations: CE: cholesterol ester; Cer: ceramide; LPS: lysophosphatidylserine; LPE: lysophosphatidylethanolamine; TG: triradylglycerols; PC: phosphatidylcholine; PE: phosphatidylethanolamine; AC: acylcarnitine; PG: phosphatidylglycerol; PI: phosphatidylinositol; O-: ether-linked lipid.

Table S5. Lipid molecules differentially expressed in plasma of EED children in negative ion data
Related to Figure 4

Lipid name	Fold change (EED/Normal)	*p* value	q value (FDR adjusted)
**LPC 22:2**	-4.1	1.09E-03	0.004
**FA 18:1; O3**	-2.9	1.89E-04	0.001
**FA 18:2; O3**	-4.2	3.23E-04	0.001
**FA 18:2; O2**	-2.8	6.75E-04	0.003
**8F6 (Furocarpic acid)**	-2.1	1.45E-02	0.032
**Glycocholic Acid (GCA)**	2.7	1.40E-03	0.005
**Glycochenodeoxycholic acid (GCDCA)**	2.1	1.85E-02	0.038
**LPE 16:0**	3.9	1.47E-06	<0.001
**LPE O-16:1**	2.3	4.53E-11	<0.001
**LPE O-18:1**	2.4	1.69E-05	<0.001
**PC 28:0**	2.1	1.78E-02	0.037
**PC 31:0**	3.2	2.67E-04	0.001
**PE 34:1**	2.2	7.90E-04	0.003
**PE 36:2**	2.5	1.18E-03	0.004
**PE 36:4**	2.2	4.32E-04	0.002
**PE 38:6**	3.2	5.96E-03	0.017
**PE O-34:1**	3.0	2.61E-07	<0.001
**PE O-34:2**	2.1	6.40E-10	<0.001
**PE O-36:5**	2.2	7.00E-04	<0.001
**PE O-36:3**	2.2	4.20E-05	<0.001
**PE O-36:4**	5.3	6.09E-08	0.003
**PE O-36:6**	2.1	6.42E-05	<0.001
**PI 34:1**	2.9	1.24E-07	<0.001
**PI 40:5**	4.8	2.39E-05	<0.001
**PI 32:1**	2.5	1.73E-05	<0.001
**PI 38:6**	2.6	1.82E-03	0.006
**PI 40:6**	4.1	1.64E-04	0.001

*27 features significantly changed between the two groups with fold change > 2 and FDR adjusted q-value < 0.05; among them 5 significantly down-regulated in AKU (represented as negative value), 22 significantly up-regulated in AKU. Fold change colored in blue indicates a decrease of EED children compared to their normal counterpart, red indicate an increase.
Lipid class abbreviations: FA: fatty acid; LPC: lysophosphatidylcholine; LPS: lysophosphatidylserine; LPE: lysophosphatidylethanolamine; PC: phosphatidylcholine; PE: phosphatidylethanolamine; PI: phosphatidylinositol; O-: ether-linked lipid.

Table S6. Lipid molecules differentially expressed in duodenal aspirate of EED children in positive ion data

Lipid name	Fold change (EED/Normal)	*p* value	q value (FDR adjusted)
Cer(d18:0/26:0)	-2.5	6.67E-06	<0.001
Cer(d18:1/26:0)	-2.7	9.00E-06	<0.001
Cer(t18:0/24:0)	-2.0	2.59E-06	<0.001
Cer(t18:0/26:0)	-2.1	5.18E-07	<0.001
Cer(d18:1/26:0(OH))	-2.4	1.64E-04	0.002
GlcCer(d18:0/20:0)	-5.3	2.68E-05	0.001
GlcCer(d18:0/22:0)	-2.8	4.71E-05	0.001
GlcCer(d18:0/26:0)	-6.1	1.14E-06	<0.001
GlcCer(t18:0/24:1)	-2.7	1.30E-03	0.012
GlcCer(d18:1/25:0(OH))	-2.7	5.62E-05	0.001
GlcCer(t18:0/22:0(OH)	-2.3	4.36E-04	0.004
LPC 20:0	-2.1	1.63E-03	0.013
LPC O-16:1	-2.8	2.75E-03	0.021
LPC O-18:1	-2.5	2.47E-03	0.019
LPC O-18:2	-3.0	7.01E-04	0.007
LPS 20:0	-2.4	1.58E-04	0.002
LPS 22:0	-3.4	5.88E-06	<0.001
SM(d18:1/15:0)	-2.9	1.07E-04	0.001
SM(d18:1/16:0)	-3.0	2.53E-04	0.003
SM(d18:1/18:0)	-2.6	4.42E-05	0.001
SM(d18:1/20:0)	-8.0	4.08E-06	<0.001
SM(d18:1/22:0)	-2.6	2.98E-05	0.001
SM(d18:1/24:0)	-2.4	7.64E-05	0.001

*23 positive ion features (fold change > 2 & FDR adjusted q-value < 0.05) between AKU and CCHMC control, all 23 significantly down-regulated in AKU (represented as negative value). Fold change colored in blue indicates a decrease of EED children compared to their normal counterpart, red indicate an increase.

Lipid class abbreviations: Cer: ceramide; GlcCer: glucosylceramide; LPC: lysophosphatidylcholine; LPS: lysophosphatidylserine; SM: sphingomyelin; O-: ether-linked lipid.

Table S7. Lipid molecules differentially expressed in duodenal aspirate of EED children in negative ion data
Related to Figure 4

Lipid name	Fold change (EED/Normal)	*p* value	q value (FDR adjusted)
Cer(d18:0/24:0)	-2.1	3.41E-03	0.033
GlcCer(d18:0/24:0)	-7.2	1.39E-05	<0.001
GlcCer(d18:0/26:0)	-10.4	3.38E-06	<0.001
LPC O-16:1	-2.5	4.39E-03	0.038
LPE(20:0)	-2.7	5.61E-03	0.045
LPE(20:4)	-5.1	6.53E-03	0.045
LPG(18:1)	-4.4	6.05E-03	0.045
LPI(18:0)	-2.3	6.62E-03	0.045
LPS(20:0)	-3.0	3.79E-04	0.005
SM 34:0	-2.3	7.53E-04	0.009
SM 32:1	-3.5	2.07E-04	0.004
SM(d18:1/16:0)	-2.1	1.33E-03	0.014
SM(d18:1/18:0)	-3.4	3.39E-05	0.001
SM(d18:1/22:0)	-2.9	3.62E-05	0.001
SM 41:1	-2.4	4.15E-03	0.038
SM(d18:1/24:0)	-2.7	7.86E-05	0.002
TLCA	-6.6	3.02E-04	0.005
FA 18:0; O2	16.0	3.90E-10	<0.001
FA 18:1; O2	3.3	5.17E-07	<0.001

*19 negative ion features (fold change > 2 & FDR adjusted q-value < 0.05) between AKU and CCHMC control, 17 significantly down-regulated in AKU (represented as negative value) while 2 up-regulated. Fold change colored in blue indicates a decrease of EED children compared to their normal counterpart, red indicate an increase.

Lipid class abbreviations: FA: fatty acid; Cer: ceramide; GlcCer: glucosylceramide; LPC: lysophosphatidylcholine; LPS: lysophosphatidylserine; LPE: lysophosphatidylethanolamine; PC: phosphatidylcholine; LPI: lyphosphatidylinositol; SM: sphingomyelin; TLCA: Taurolithocholic acid; O-: ether-linked lipid.

Table S8. Sphingomyelin (SM) that were differentially expressed in duodenal aspirate of EED children showed a negative correlation to composite EED histological score
Related to Figure 4

Lipid ID	Correlation coefficient to composite EED score
SM (d18:1/15:0)	-0.123
SM (d18:1/16:0)	-0.114
SM (d18:1/18:0)	-0.124
SM (d18:1/20:0)	**-0.289**
SM (d18:1/22:0)	-0.070
SM (d18:1/23:0)	-0.102
SM (d18:1/24:0)	-0.082
SM (d18:1/24:1)	-0.073
Total SM	-0.155

*Correlation coefficient with P<0.05 are highlighted in bold

Supplemental Methods:

Table S9: Environmental Enteric Dysfunction (EED) Scoring, related to Figure 5

Feature	Grade	Description
**Description**		EED scoring includes assessment of the following features: acute inflammation (score 0-3), eosinophil infiltration (score 0-3), chronic inflammation in the lamina propria (score 0-3), intra-epithelial lymphocytes (score 0-4), villus architecture (score 0-4), intramucosal brunner glands (score 0-3), foveolar cell metaplasia (score 0-3), goblet cell density (score 0-3), Paneth cell density (score 0-3), enterocyte injury (score 0-3), and epithelial detachment (score 0-4).
**Feature**	**Grade**	**Description**
Acute inflammation	0	No PMNs observed, or only PMNs in lamina propria with no infiltration of epithelium by PMNs (cryptitis, villitis)
	1	1-2 foci of epithelial PMN infiltration or crypt microabscesses
	2	> 2 foci of epithelial PMN infiltration or crypt microabscesses but <50% of mucosa involved
	3	≥ 50% of mucosa involved by epithelial PMN infiltration
Eosinophil infiltration	0	No increase in eosinophils (highly scattered in lamina propria, no intravillus or intercryptal space with >5 eosinophils)
	1	Increased eosinophils (intravillus or intercryptal space with >5 eosinophils) involving < 50% of mucosa, with no eosinophilic crypt microabcesses
	2	Increased eosinophils (intravillus or intercryptal space with >5 eosinophils) involving > 50% of mucosa, or up to 1 focus of eosinophilic epithelial infiltration or crypt microabcesses per mucosal fragment
	3	>2 foci of eosinophilic epithelial infiltration or crypt microabcesses in any mucosal fragment
Chronic inflammation-lamina propria	0	No qualitative increase in mononuclear inflammatory cells (MIC) in lamina propria. Majority of villus bases contain ≤3 MIC across, on average.
	1	Increased MIC, based on villus base displaying 3-5 MIC across, on average.
	2	Increased MIC, based on villus base displaying 6-10 MIC across, on average.
	3	Increased MIC, based on villus base displaying >10 lymphocytes on average.
Intra-epithelial lymphocytes	0	No areas observed with epithelial/lymphocyte ratio ≥20%
	1	Lymphocyte/epithelial ratio ≥20%, but <50%, in less than 50% of mucosa
	2	Lymphocyte/epithelial ratio ≥20%, but <50%, in greater than 50% of mucosa
	3	Lymphocyte/epithelial ratio ≥50% in less than 50% of mucosa
	4	Lymphocyte/epithelial ratio ≥50% in greater than 50% of mucosa
Villus architecture	0	Majority of villi are >3 crypt lengths long
	1	Villi are ≤ 3 but > 2 crypt lengths long, in < 50% of mucosa.
	2	Majority of villi are ≤ 2 crypt lengths long, but > 1 crypt length long
	3	Villi absent, or ≤1 crypt length long, in < 50% of mucosa.
	4	Villi absent, or <1 crypt length long, in > 50% of mucosa.
Intramucosal Brunner glands	0	None observed
	1	One or two foci, none involving more than 5 crypt bases
	2	3-5 foci, none involving more than 5 crypt bases
	3	> 5 foci, or any area of intramucosal Brunner glands involving >5 crypt bases
Foveolar cell metaplasia	0	Not observed
	1	1-2 villus tips involved
	2	3-5 villus tips involved
	3	> 5 villus tips involved
Goblet cell density	0	Most villi contain ≥10 goblet cells
	1	Goblet cells <10/ villus, involving < 25% of mucosa
	2	Goblet cells <10/ villus, involving 25-50% of mucosa
	3	Goblet cells <10/ villus, involving >50% of mucosa
Paneth cell density	0	≥5 Paneth cells/ crypt, on average
	1	2-4 Paneth cells/ crypt, on average
	2	<2 Paneth cell/crypt, involving <50% of crypt bases
	3	<2 Paneth cell/crypt, involving >50% of crypt bases
Enterocyte injury	0	Majority of enterocytes (90%) show tall columnar morphology
	1	Enterocytes show low columnar (≤2:1 L:W ratio), cuboidal or flat morphology, in < 50% of mucosa

	2	Enterocytes show low columnar (≤2:1 L:W ratio), cuboidal or flat morphology, in > 50% of mucosa
	3	Any area of mucosal erosion/ulceration
Epithelial detachment	0	Complete coverage of mucosal surface by epithelial cells
	1	Surface epithelium missing or detached from <25% of mucosa
	2	Surface epithelium missing or detached from 25-50% of mucosa
	3	Surface epithelium missing or detached from 51-75% of mucosa
	4	Surface epithelium missing or detached from >75% of mucosa
